# Radiomics-Based Machine Learning Technology Enables Better Differentiation Between Glioblastoma and Anaplastic Oligodendroglioma

**DOI:** 10.3389/fonc.2019.01164

**Published:** 2019-11-05

**Authors:** Yimeng Fan, Chaoyue Chen, Fumin Zhao, Zerong Tian, Jian Wang, Xuelei Ma, Jianguo Xu

**Affiliations:** ^1^Department of Ophthalmology, West China Hospital, Sichuan University, Chengdu, China; ^2^State Key Laboratory of Biotherapy and Cancer Center, West China Hospital, Sichuan University, Chengdu, China; ^3^Department of Neurosurgery, West China Hospital, Sichuan University, Chengdu, China; ^4^Department of Radiology, West China Second University Hospital, Sichuan University, Chengdu, China; ^5^School of Computer Science, Nanjing University of Science and Technology, Nanjing, China; ^6^Department of Biotherapy, Cancer Center, West China Hospital, Sichuan University, Chengdu, China; ^7^State Key Laboratory of Biotherapy and Cancer Center, West China Hospital, Sichuan University, and Collaborative Innovation Center for Biotherapy, Chengdu, China

**Keywords:** machine learning, magnetic resonance imaging, glioblastoma, anaplastic oligodendroglioma, texture analysis

## Abstract

**Purpose:** The aim of this study was to test whether radiomics-based machine learning can enable the better differentiation between glioblastoma (GBM) and anaplastic oligodendroglioma (AO).

**Methods:** This retrospective study involved 126 patients histologically diagnosed as GBM (*n* = 76) or AO (*n* = 50) in our institution from January 2015 to December 2018. A total number of 40 three-dimensional texture features were extracted from contrast-enhanced T1-weighted images using LIFEx package. Six diagnostic models were established with selection methods and classifiers. The optimal radiomics features were separately selected into three datasets with three feature selection methods [distance correlation, least absolute shrinkage and selection operator (LASSO), and gradient boosting decision tree (GBDT)]. Then datasets were separately adopted into linear discriminant analysis (LDA) and support vector machine (SVM) classifiers. Specificity, sensitivity, accuracy, and area under curve (AUC) of each model were calculated to evaluate their diagnostic performances.

**Results:** The diagnostic performance of machine learning models was superior to human readers. Both classifiers showed promising ability in discrimination with AUC more than 0.900 when combined with suitable feature selection method. For LDA-based models, the AUC of models were 0.986, 0.994, and 0.970 in the testing group, respectively. For the SVM-based models, the AUC of models were 0.923, 0.817, and 0.500 in the testing group, respectively. The over-fitting model was GBDT + SVM, suggesting that this model was too volatile that unsuitable for classification.

**Conclusion:** This study indicates radiomics-based machine learning has the potential to be utilized in clinically discriminating GBM from AO.

## Introduction

High-grade gliomas, the most common malignant solidary brain tumors in adults, are traditionally classified into anaplastic oligodendroglioma (AO), anaplastic astrocytoma (AA), and Glioblastoma (GBM) (1, 2). According to the WHO classification, AO was ascribed in Grade III while GBM was ascribed in Grade IV based on their histology characteristics. The early diagnosis of GBM and AO is clinically challenging but necessary due to their different treatment choice as well as the therapeutic responsiveness and patient survival (3). As for GBM extended resection is recommended to increase patient survival, whereas for AO this strategy lacks solid evidence (4–6). The treatment after surgery is also different as well. For GBM, standardized therapy after surgery recommended by NCCN guidelines is standard brain radiation therapy (RT) + concurrent temozolomide (TMZ) followed by adjuvant chemotherapy (7). While for AO, it is recommended to use fractionated external beam RT together with neoadjuvant or adjuvant PCV (procarbazine, lomustine, and vincristine) regarding the specific condition of each patient (7, 8).

A glioma-specific blood biomarker for glioma has not been identified yet. Therefore, the radiology examination is critical for tumor detection and lesion localization. Brain magnetic resonance imaging (MRI) plays a key role in the preoperative diagnostic of gliomas with high image resolution on tumor tissue. However, in some cases, MRI may be unable to provide enough information for differentiation between GBM and AO. The MRI characteristics of two tumors are pretty similar when GBM is characterized by perilesional vasogenic edema and ring-like enhancement (9–11); while AO also shows peritumoral edema and heterogeneous enhancement (11, 12). In this regard, the urgency of new radiological method has been highlighted.

Given that texture analysis on images provides a more objective information beyond naked eye assessment, quantitative descriptions of tumor characteristics could be an option for clinical diagnosis (13–16). Moreover, with digital parameters, new technology, such as machine learning, can be introduced for further statistical analysis. Machine learning, a hotspot in the field of artificial intelligence, enables the extraction of meaningful patterns from massive datasets and thereby achieving precise predictions with the model built (17). Machine learning has demonstrated outstanding performance in previous research including segmentation of the tumor, classification of certain types of tumor, and prediction of survival or genotype (18–23). Although the differentiation between GBM and AO is of high clinical relevance, the machine learning approach has never been explored yet. In this study, we investigated the feasibility of radiomics-based machine learning to differentiate GBM and AO.

## Materials and Methods

### Study Patients

In this retrospective single-center research, we viewed medical records in neurosurgery department to initially search for patients histologically diagnosed with GBM or AO from January 2015 to December 2018. The medical records were reviewed by two researchers to enroll the potentially qualified patients and to collect relevant clinical information for our research. The inclusion criteria for patients were: (1) with pathological diagnosis of GBM or AO in intraoperative freezing biopsy, and (2) with available high-quality pre-treatment MR scan performed at our institution before surgical resection. Then the pre-surgical MRI images of patients were exported from radiological department though Picture Archiving and Communication Systems (PACS) with uniform standard.

For patients before 2016, we made correction on their pathological diagnoses based on the new World Health Organization 2016 classification of gliomas by a senior neuropathologist with working experience of 10 years (24). The new standards required the presence of both IDH-mt and 1p19q co-deletion for the diagnosis of AO, otherwise it could only be regarded as NOS (Not Otherwise Specified) (24). Therefore, we excluded patients based on new classification who were with incomplete gene reports or with absent presence of both gene expression.

Seventy-nine consecutive patients with GBM and 56 consecutive patients with AO fulfilled inclusion criteria in the initial selection. Three patients with GBM and six patients with AO were excluded in the following evaluation according to the exclusion criteria, which were: (1) presence of motion artifacts on MRI, (2) previous history of brain surgery or biopsy, (3) previous history of intracranial diseases, such as subarachnoid hemorrhage, cerebral infarction, etc. Based on this strategy, a study cohort was built consisting of 76 GBM patients (mean age: 46.5 years) and 50 AO patients (mean age: 47.1 years).

All procedures performed in studies involving human participants were in accordance with the ethical standards of the institutional and/or national research committee and with the 1964 Helsinki declaration and its later amendments or comparable ethical standards. The institutional review board approved this retrospective study. The written informed consent was obtained from participants enrolled in this study. The written informed consent was necessary before radiological examination (written informed consent for patients <16 years old was signed by parents or guardians) for each patient. The patients agreed to undertake examination when needed and were informed that the statistics (including MR image), which could be used for academic purpose in the future, would be stored in our institutional database. The Ethics Committee of Sichuan University and radiology department of our institution have approved for statistics export and utilization for this study.

### MR Image Acquisition

The current study focused on the conventional MR sequences. The suitable sequence should be chosen first for two reasons, that the descriptions on features boundary were vague in some sequences, and that features from all sequences would bring too much burden on classifiers. After initial evaluation on images and consultation with senior radiologists, the contrast-enhanced MRI sequence was the only one used to perform texture analysis in this study.

**Figure 1:**
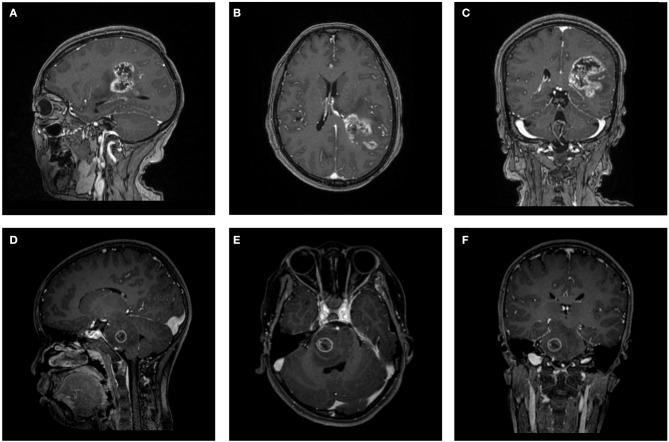
Two examples of contrast-enhanced MRI images. **(A–C)** Patient with GBM in **(A)** parasagittal, **(B)** axial, and **(C)** coronal view. **(D–F)** Patient with AO in **(D)** parasagittal, **(E)** axial, and **(F)** coronal view. GBM, glioblastoma; AO, anaplastic oligodendroglioma.

The MR scans were performed in the radiology department of institution. The contrast-enhanced MRI sequences were obtained with a 3.0T Siemens Trio Scanner using a MPRAGE sequence with the following imaging parameters: TR/TE/TI = 1900/2.26/900 ms, Flip angle = 9°, slice thickness = 1 mm, axial FOV = 25.6 × 25.6 cm^2^ and data matrix = 256 × 256. Intravenous injection of gadopentetate dimeglumine (0.1 mmol/Kg) was taken as contrast agent for patients. Multi-directional data for contrast-enhanced MRI were collected during the interval time of 90-250s. Figure 1 shows two examples of contrast-enhanced MRI images.

### Human Readers Assessment

To test whether machine learning could outperform human readers, the diagnostic performance of them was compared. A senior neurosurgeon and a senior radiologist independently made diagnosis based on contrast-enhanced T1-weighted images, which were presented randomly, regarding classification as GBM or AO. Both readers were blinded to patient information and pathology reports. Then, the accuracy, sensitivity, and specificity were calculated for further analysis.

### Texture Feature Extraction

The texture features of tumor tissue were extracted by two researchers using the LIFEx package under the supervision of a senior radiologist (25). Disagreements between researchers were recorded and adjudicated by consulting senior radiologists and neurosurgeons. The volume of interest (VOI) was drawn on T1C images by contouring the outer margin of tumor tissues slice by slice. The peritumoral edema band and adjacent structure invasion were separated from the primary tumor with the difference in contrast enhancement. For the lesions with multiple (>2) enhancement foci, ROI was only performed on the biggest one for those with clear boundary, and on tumor-confirmed area for those with vague boundary. After the ROI delineation, texture features were calculated automatically with default setting.

A total of 40 three-dimensional (3D) texture features were calculated from two orders. In the first order, texture features were calculated from shape histogram-based matrix and histogram-based matrix. In the second or higher order, features were calculated from gray-level co-occurrence matrix (GLCM), gray-level zone length matrix (GLZLM), neighborhood gray-level dependence matrix (NGLDM), and gray-level run length matrix (GLRLM). To avoid the interference of the lower image matrix resolution, texture analysis performed only on the VOIs with more than 64 voxels by default setting. All original data about extracted features were shown in Supplementary Material 1.

To ensure the validity and reproducibility of the extraction, the procedure was performed twice, and the difference between two sets was examined with Manny-Whitney *U*-test. We adjusted the *q* < 0.01 as significant (before was *p* < 0.05) to avoid the interference of false-positive errors rising from a large number of texture features. The results suggested that none of the features were significantly different, implying that the results could be considered reliable and reproducible (shown in Supplementary Material 2).

### Classification Model Establishment

The purpose of machine learning was to train the models to predict whether each tumor was a GBM or AO with radiomics parameters extracted from the tumor tissue image. However, feature selection was necessary to eliminate statistically insignificant features and to avoid overfitting, which contributes to decreased running time and increased accuracy of the resulting models (26–28). In this study, we employed three selection methods with different selection mechanisms: distance correlation as representative of filter models, least absolute shrinkage and selection operator (LASSO) and gradient boosting decision tree (GBDT) as representatives of embedded models. Then, three datasets were generated with three different selection methods, which were each classified separately. The list of features selected with three different methods are shown in Supplementary Material 3, and the explanation of the features are summarized in Supplementary Material 4.

The next step was to choose suitable classifiers. Since linear classifier and non-linear classifier represent the state-of-the-art in pattern recognition, we adopted linear discriminant analysis (LDA) and support vector machine (SVM) classification algorithms in the current study as representatives of two classifier types (29). This way, overall six diagnostic models were established based on three selection methods and two classifiers.

**Figure 2:**
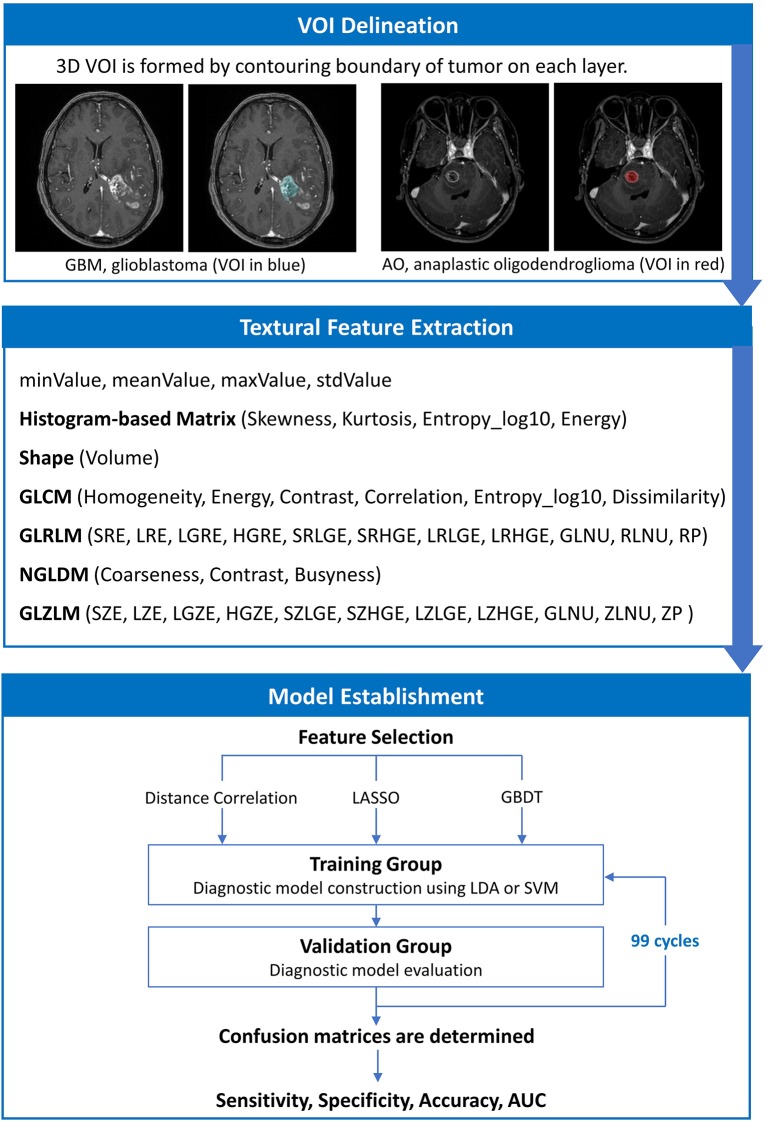
The schematic workflow from image processing to machine learning.

As for the algorithm deployment, the study cohort was randomly divided into two subsets as training group and validation group on a proportion of 4:1. When the training on classifiers finished, the validation group was fed to evaluate the diagnostic performance of the models. Sensitivity, specificity, accuracy, and area under receiver operating characteristic curve (AUC) were calculated for both the training and validation group. To appraise the robustness of the methods, the procedure was repeated for 100 cycles with different and independent case assignments. The schematic workflow from image processing to machine learning is shown in Figure 2.

## Results

### Patient Characteristics

Among 126 patients were enrolled in the current study, 76 patients were diagnosed with GBM, and 50 patients with AO. The sex ratio, mean age, and time between MR scan and pathological diagnosis were summarized in Table 1. As for the human reader assessment, the accuracy for the neurosurgeon was 63.49%, and for the radiologist was 66.77%. Based on the results, a strong tendency on misdiagnosis of AO could be observed.

**Table 1:** Demographics of patients.

	**Number**	**Sex**	**Age, mean (range), y**	**Time between MR scan and pathological diagnosis**	**Human reader 1 accuracy**	**Human reader 2 accuracy**
GBM	76	47% male, 53% female	46.5 (15–80)	6.5 days	81.58%	85.53%
AO	50	50% male, 50% female	47.1 (16–76)	7.9 days	36.00%	38.00%
All patients	126	48% male, 52% female	46.8 (15–80)	7.1 days	63.49%	66.77%

*GBM, glioblastoma; AO, anaplastic oligodendroglioma*.

### Diagnostic Performance of Models

The classification models exhibited promising discriminative ability when combined with suitable selection methods. For LDA-based models, all three models presented feasible performance with the AUC in the validation groups of 0.986, 0.994, and 0.970, respectively. For the SVM-based models, the models showed feasible performance with the AUC in the training groups of 0.923, 0.817, and 0.500. Overfitting was observed in one SVM-based model (SVM + GBDT), suggesting this model was volatile in application. The value of average sensitivity, specificity, accuracy, and AUC of training group and testing group are summarized in Table 2.

**Table 2:** Results of the discriminative model in distinguishing GBM from AO in the training and validation group.

**Classifier**	**Selection Method**	**Training group**				**Validation group**			
		**AUC**	**Accuracy**	**Sensitivity**	**Specificity**	**AUC**	**Accuracy**	**Sensitivity**	**Specificity**
LDA	Distance correlation	0.992	0.994	0.994	0.990	0.986	0.988	0.993	0.982
	LASSO	0.997	0.997	0.993	0.998	0.994	0.992	0.980	0.995
	GBDT	0.969	0.963	0.916	0.994	0.970	0.962	0.907	0.992
SVM	Distance correlation	0.922	0.938	1.000	0.906	0.923	0.938	1.000	0.910
	LASSO	0.831	0.868	0.972	0.826	0.817	0.831	0.935	0.798
	GBDT (over-fitting)	1.000	1.000	1.000	1.000	0.500	0.623	0.935	0.798

*GBM, glioblastoma; AO, anaplastic oligodendroglioma; AUC, area under curve; LDA, linear discriminant analysis; LASSO, least absolute shrinkage and selection operator; GBDT, gradient boosting decision tree; SVM, support vector machine*.

**Figure 3:**
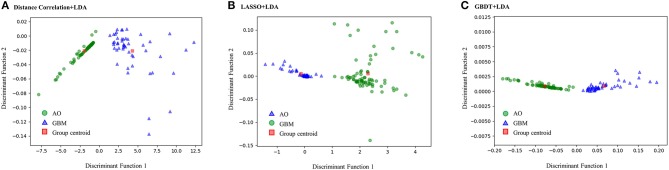
Relationships between the canonical discriminant functions for GBM, AO and the group centroids. The two distinctive clusters formed by GBM and AO suggest three LDA-based models have excellent discriminant ability for GBM and AO. GBM, glioblastoma; AO, anaplastic oligodendroglioma; LDA, linear discriminant analysis; LASSO, least absolute shrinkage and selection operator; GBDT, gradient boosting decision tree; SVM, support vector machine. **(A)** Canonical discriminant functions for Distance Correlation + LDA. **(B)** Canonical discriminant functions for LASSO + LDA. **(C)** Canonical discriminant functions for GBDT + LDA.

**Figure 4:**
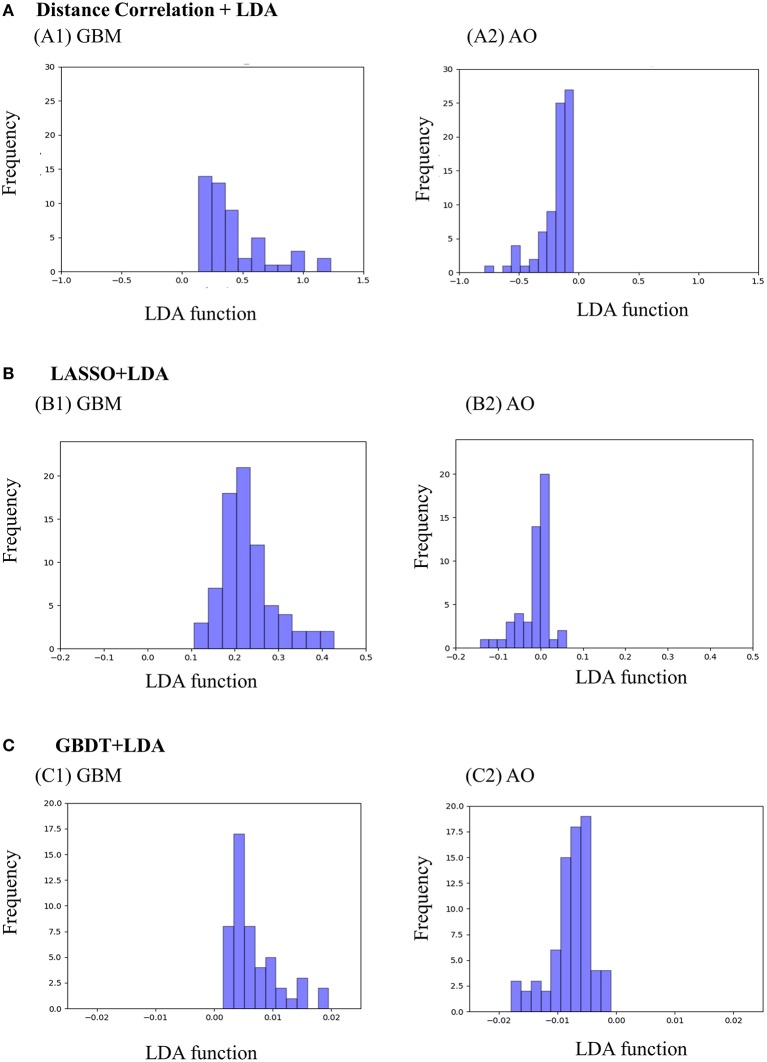
Examples of performance of the LDA-based models in terms of the distribution of the canonical functions determined for the GBM and AO for two of the 100 independent training cycles in the MRI analysis. Minimal overlap is observed, suggesting high differential ability of the models. **(A)** Distance Correlation + LDA, **(B)** LASSO + LDA, **(C)** GBDT + LDA. GBM, glioblastoma; AO, anaplastic oligodendroglioma; AUC, area under curve; LDA, linear discriminant analysis; LASSO, least absolute shrinkage and selection operator; GBDT, gradient boosting decision tree.

Figure 3 represents the two-dimensional projection of the LDA-based models, illustrating that the GBM and AO formed distinctive clusters in the space defined by discriminant functions 1 and 2 generated by LASSO + LDA. Figure 4 shows the examples of performance of LDA-based models in terms of the distribution of the canonical functions in the 100 independent training cycles in the MRI analysis. A clear negative-values shift of the LDA function can be observed for AO, and all positive-values shift for GBM. ROCs of all models are shown in Supplementary Material 5.

## Discussion

For patients with high-grade gliomas, accurate tumor classification is clinically important because of its close relation with treatment strategy as well as therapeutic responsiveness and prognosis (3). In this study, we applied radiomics-based machine learning to pre-surgically differentiate between GBM and AO. Six models based on three selection methods (distance correlation, LASSO, and GBDT) and two classifiers (LDA and SVM) were built and evaluated. Our results demonstrated that machine learning approaches can be utilized and are clearly superior to human reader diagnosis.

Previous studies have explored the possibility of using machine learning for classification of brain tumor types (18, 21, 30). In the setting of gliomas, several studies have proved the utility of machine learning to differentiate between high-grade and low-grade glioma with high accuracy (0.80 and 0.945) (31, 32). In the setting of differentiation among specific histological subtypes of gliomas, a computer-aided diagnosis system was proposed and evaluated in a previous study to distinguish GBM from lower-grade gliomas, with positive results (33). A multicenter investigation also confirmed the feasibility of using 3D texture analysis for pediatric glioma subtype classification (medulloblastoma, pilocytic astrocytoma, and ependymoma) with an overall accuracy of 0.87 (34). The current study investigated a subject that has never been explored before, that the feasibility of radiomics-based machine learning in discriminating GBM from AO. Diagnostic performance of six models was assessed in the current study built on three selection methods (distance correlation, LASSO, and GBDT) coupled with two classifiers (LDA and SVM). In general, both classifiers showed high diagnostic performance with AUC more than 0.900 when combined with a suitable selection method. Nevertheless, when comparing between two classifiers, LDA-based models had slightly better diagnostic performance than that of SVM-based models.

The diagnostical models were established based on two types of classifies which differ in computing mechanism considering the performance of a certain classifier may be various in the settings of different tumors. LDA is a representative of the linear classifier which uses a straight line (a vector) to separates two classes (GBM and AO in this case), while SVM, a representative of the non-linear classifier, uses so-called support vectors to define a polynomic hyperplane to separate classes (35). In the settings of differentiating GBM and AO, our results showed LDA-based models had slightly better diagnostic performance than that of SVM-based models. However, the difference between the models was too slight to select the superior one, specifically given that all models investigated seemed to perform quite comparably and variance in AUC might be partially attributed due to the small statistical group. Therefore, limited by the small study cohort and relatively complicated methods, our results could only be regarded as hypothesis generation for future larger studies.

The results also implied that feature selection methods have impacts on diagnostic performance, especially for SVM-based models. Current feature selection methods can be categorized into three types depending on their selection mechanism: (1) Filter models select features by ranking them based on certain general characteristics such as correlation to remove irrelevant features without using any machine-learning algorithms. (2) Wrapper Models utilize a specific classifier to evaluate the quality of selected features, and offer a simple and powerful way to address the problem of feature selection, regardless of the chosen learning machine. (3) Embedded models are similar to wrapper models but embeds feature selection with classifier construction. Such models have the advantages of wrapper models-they include the interaction with the classification model, while embedded models are far less computationally intensive than wrapper models (28). In this study, we employed three selection methods as representatives of different selection mechanisms: distance correlation as representative of filter models, LASSO, and GBDT as representatives of embedded models. There was a common set of features selected by all three selection methods or two of the methods, which suggested these features might be important for the classification. For other features, it is hard to tell what extent they influenced the algorithms, since the AUCs showed minimal difference. However, even with feature selection, overfitting was still observed in one model (GBDT + SVM). We are unable to provide the exact reason but hypothesis that this model might be overly complex to be used as a discriminative tool to differentiate between GBM and AO.

Besides the comparison between machine learning models, we also performed comparison between machine and human readers in this study. Two readers unaware of the information on the exact number of patients were asked to make diagnosis on GBM or AO based on MR images. The readers were chosen from neurosurgery department or radiology department to ensure the convincing and reliable conclusions. The results were unexpected, considering there were only two options, that the diagnostical accuracy on AO was even lower than 0.500. Specifically, AO was easily misdiagnosed as GBM in human readers' radiological assessments. The explanations from the readers were the same that GBM and AO usually represented similar patterns on MR images, and they prefer to choose GBM rather than AO in these cases due to the epidemiological reason that the incidence of GBM is much higher than AO. As we mentioned before, the accurate pre-surgical diagnosis for two types of tumor is clinical important given the differences in surgical strategy. Therefore, it is reasonable to draw the conclusion that the patients will benefit from better treatment with machine learning clinical assistances. Machine intelligence will urge the radiological practice to change dramatically. However, we should also realize that the current machine technology is far from replacing human readers, and a combination of radiologist and machine might be the best choice for the foreseeable future. Radiologists still lead the central role in diagnosis while machine only act as assistance. This combination virtually eliminates simple blunders, increases play level, and provides better insight into the decision process (36).

Our study has several limitations. Firstly, it was a retrospective single-center investigation, which may lead to a patient selection bias and limited sample size. However, at present stage, it is still unknowable how much data is required to establish a predictive model, which may be answered through empirical investigation. The number of patients enrolled in previous studies focusing on similar topic ranged from 25 to 534 (31–34, 37–39). Secondly, we did not perform subgroup analysis regarding the IDH mutation status of GBM patients. Recent studies reported machine-learning based MRI texture analysis could be used as a new method for prediction of IDH mutational status, which suggested that IDH mutational status might have bearing on texture features (37, 39, 40). Thirdly, we used conventional contrast-enhanced MRI images only and did not use other sequences or advanced imaging tools such as magnetic resonance spectroscopy (MRS). Contrast-enhanced MRI sequence was chosen in this study for the clear delineation of tumor boundaries. The combined use of other sequences or imaging tools may enable better diagnostic ability. Fourthly, models built in current study were not externally validated. Since medical centers use different MRI scanners, imaging parameters and contrast, radiomic features may change accordingly. Therefore, the efficacy of machine learning-based models in this study cannot be guaranteed for external datasets. Nevertheless, we used the open-source package to perform the image processing and texture analysis, which allows others to reproduce the texture analysis with other datasets.

## Conclusion

In conclusion, radiomics-based machine learning enables differentiation between glioblastoma and anaplastic oligodendroglioma. Our data indicate that the performance of this approach is superior to a human reader. This method may be a valuable addition to routine clinical practice to improve GBM and AO differentiation. However, multicenter investigations including larger patient cohorts and analysis combined with other MRI sequences or imaging techniques are warranted so that this non-invasive approach can be introduced into routine clinical practice.

## Data Availability Statement

We are pleased to share our data to any qualified researchers without undue reservation. Please contact corresponding author if there is anything they need.

## Ethics Statement

The studies involving human participants were reviewed and approved by Ethics Committee of Sichuan University. Written informed consent to participate in this study was provided by the participants' legal guardian/next of kin. Written informed consent was obtained from the individual(s), and minor(s)' legal guardian/next of kin, for the publication of any potentially identifiable images or data included in this article.

## Author Contributions

YF, CC, JX, and XM contributed conception and design of the study. CC and ZT enrolled eligible patients, obtained medical records, and MRI images of each patient. FZ and CC independently made diagnosis based on contrast-enhanced T1-weighted images. YF and ZT did texture analysis. JW established the models and performed other statistical analysis. YF wrote the first draft of the manuscript. CC wrote sections of the manuscript. All authors contributed to manuscript revision, read, and approved the submitted version.

### Conflict of Interest

The authors declare that the research was conducted in the absence of any commercial or financial relationships that could be construed as a potential conflict of interest.

## Supplementary Material

Supplementary Material 1 Original data of extracted features. LASSO, least absolute shrinkage and selection operator; GBDT, gradient boosting decision tree.

Supplementary Material 2 Results of Manny-Whitney *U*-test to examine the difference between two sets of data extraction.

Supplementary Material 3 Texture features selected with three different methods. Features selected by all three methods are labeled red. Features selected by two methods are labeled in yellow. LASSO, least absolute shrinkage and selection operator; GBDT, gradient boosting decision tree.

Supplementary Material 4 Explanation of each texture features.

Supplementary Material 5 ROCs of six models to differentiate between GBM and AO. GBM, glioblastoma; AO, anaplastic oligodendroglioma; LASSO, least absolute shrinkage and selection operator; GBDT, gradient boosting decision tree; LDA, linear discriminant analysis; SVM, support vector machine.

## Abbreviations

AO: anaplastic oligodendroglioma
AUC: area under curve
GBDT: gradient boosting decision tree
GBM: glioblastoma
LASSO: least absolute shrinkage and selection operator
LDA: linear discriminant analysis
MRI: magnetic resonance imaging
SVM: support vector machine
T1C image: contrast-enhanced T1-weighted image
VOI: volume of interest.
