# Supplementary material for: Radiomics-Based Machine Learning Technology Enables Better Differentiation Between Glioblastoma and Anaplastic Oligodendroglioma
Source: Front Oncol. 2019 Nov 5;9:1164. doi: 10.3389/fonc.2019.01164 (PMC6848260; doi:10.3389/fonc.2019.01164)
Supplement: Supplementary Material 1 — Original data of extracted features. LASSO, least absolute shrinkage and selection operator; GBDT, gradient boosting decision tree. [file Data_Sheet_1.PDF]

Patient	1	2	3	4	5	6	7	8	9	10	11
Type(GBM=2, AO=1)	2	2	2	2	2	2	2	2	2	2	2
minValue	0	0	0	54	0	11	52	278	110	1310	1260
meanValue	180	94.3	73.5	316	127	752	766	576	298	3110	3610
stdValue	87.5	71.4	51.3	115	44.6	256	335	94.8	72.2	758	1200
maxValue	503	400	236	1250	534	1740	2260	899	599	6490	8730
HISTO_Skewness	0.581	1.23	0.574	1.07	0.891	0.335	1.23	-0.294	0.434	0.883	0.663
HISTO_Kurtosis	3.06	3.82	2.54	4.55	5.48	2.84	4.59	2.6	3.22	3.93	3.17
HISTO_Entropy_log10	1.53	1.28	1.26	1.61	1.23	2	2.06	1.57	1.46	1.3	1.02
HISTO_Energy	0.0346	0.082	0.0613	0.0298	0.0754	0.0119	0.0111	0.0312	0.0412	0.265	0.428
SHAPE_Volume (mL)	8.83	105	20.5	93.3	192	42.5	84.8	9.78	17.1	54.6	44
GLCM_Homogeneity	0.265	0.429	0.409	0.281	0.482	0.148	0.17	0.242	0.365	0.37	0.506
GLCM_Energy	0.00246	0.0183	0.0119	0.00251	0.014	0.000253	0.000384	0.00194	0.00486	0.106	0.229
GLCM_Contrast	68.9	30.6	29.4	162	14.7	443	994	126	41.4	1080	1620
GLCM_Correlation	0.516	0.598	0.363	0.435	0.533	0.645	0.591	0.152	0.575	0.522	0.432
GLCM_Entropy_log10	2.76	2.24	2.17	3.02	2.16	3.79	3.82	2.88	2.61	2.53	1.98
GLCM_Dissimilarity	6.18	3.44	3.84	8.55	2.52	15.4	21	8.21	4.56	21	23
GLRLM_SRE	0.96	0.887	0.874	0.94	0.833	0.981	0.972	0.961	0.905	0.868	0.807
GLRLM_LRE	1.19	1.73	2.84	1.46	3.31	1.08	1.16	1.2	1.97	5.47	9.01
GLRLM_LGRE	0.0000721	0.0000827	0.0000858	0.0000579	0.0000782	0.0000345	0.0000346	0.0000405	0.0000592	0.0000076	7.86E-06
GLRLM_HGRE	14200	12300	11800	17800	12900	31600	32700	25100	17100	137000	135000
GLRLM_SRLGE	0.0000691	0.0000728	0.0000747	0.0000542	0.000065	0.0000338	0.0000335	0.0000389	0.0000536	6.77E-06	6.64E-06
GLRLM_SRHGE	13600	11000	10300	16800	10700	31000	32000	24100	15500	116000	104000
GLRLM_LRLGE	0.0000864	0.000148	0.000249	0.0000875	0.000259	0.0000373	0.000041	0.0000484	0.000118	0.0000354	0.0000577
GLRLM_LRHGE	16700	20600	32600	25000	42500	33900	36900	30100	33400	854000	1420000
GLRLM_GLNU	65.1	1600	724	1750	7810	526	607	206	434	1260	1860
GLRLM_RLNU	1730	17900	10000	53400	81900	42200	52200	6100	9070	7140	5240
GLRLM_RP	0.947	0.842	0.824	0.91	0.778	0.975	0.96	0.948	0.862	0.73	0.621
NGLDM_Coarseness	0.00412	0.000255	0.000455	0.0000916	0.0000623	0.000221	0.000129	0.000943	0.000752	0.0000251	0.000015
NGLDM_Contrast	0.24	0.158	0.252	0.169	0.028	0.492	0.677	0.286	0.136	1.27	1.63
NGLDM_Busyness	0.282	10.8	17.9	3.17	15.3	0.373	0.487	0.511	1.28	1.18	1.59
GLZLM_SZE	0.711	0.632	0.443	0.667	0.432	0.818	0.788	0.682	0.539	0.906	0.931
GLZLM_LZE	9.43	7830	3050	670	135000	3.02	8.51	10.1	518	8200	18500
GLZLM_LGZE	0.0000699	0.0000758	0.0000814	0.0000545	0.0000764	0.0000341	0.0000323	0.0000409	0.0000579	8.19E-06	8.97E-06
GLZLM_HGZE	14600	13400	12400	18900	13300	32100	35200	24900	17500	126000	117000
GLZLM_SZLGE	0.0000489	0.0000474	0.0000359	0.0000358	0.0000323	0.0000277	0.0000248	0.000028	0.0000309	7.39E-06	8.34E-06
GLZLM_SZHGE	10500	8560	5520	12900	5870	26500	28500	16900	9520	115000	109000
GLZLM_LZLGE	0.000717	0.714	0.285	0.0465	10.7	0.000108	0.000359	0.000401	0.0308	0.051	0.115
GLZLM_LZHGE	126000	85900000	32700000	9670000	1.7E+09	90500	216000	259000	8740000	1.32E+09	2.98E+09
GLZLM_GLNU	32	240	72.1	657	255	362	336	95.1	71.1	57.5	35
GLZLM_ZLNU	479	2300	249	10800	1100	20400	20900	1510	597	4600	3680
GLZLM_ZP	0.509	0.221	0.0879	0.389	0.0424	0.719	0.621	0.496	0.173	0.428	0.317

Patient	12	13	14	15	16	17	18	19	20	21	22
Type(GBM=2, AO=1)	2	2	2	2	2	2	2	2	2	2	2
minValue	121	34	475	187	222	0	217	299	7	180	140
meanValue	333	641	2320	513	4420	252	410	941	2890	323	507
stdValue	89.5	137	631	80	750	56.2	82.2	222	997	45.3	152
maxValue	800	1250	6010	850	7640	765	1030	1480	6350	572	1030
HISTO_Skewness	0.35	-1.48	0.627	-0.00292	-0.785	0.635	0.471	-0.481	0.135	-0.0567	0.341
HISTO_Kurtosis	3.41	6.57	4.07	3.52	4.51	5.8	3.7	2.96	2.56	3.72	2.78
HISTO_Entropy_log10	1.56	1.67	2.12	1.51	0.168	1.35	1.51	1.93	1.53	1.26	1.78
HISTO_Energy	0.0325	0.0296	0.0231	0.0389	0.916	0.0538	0.0346	0.0135	0.218	0.0673	0.0195
SHAPE_Volume (mL)	50.2	38.2	130	86.4	117	59.5	78.8	13.1	88	54.5	20.3
GLCM_Homogeneity	0.282	0.305	0.156	0.337	0.957	0.349	0.267	0.138	0.345	0.403	0.251
GLCM_Energy	0.00196	0.0024	0.00547	0.00337	0.91	0.00454	0.00197	0.000361	0.0991	0.00854	0.000999
GLCM_Contrast	88.6	141	1990	52.4	225	29.3	86.1	852	4610	18.2	137
GLCM_Correlation	0.492	0.538	0.6	0.556	0.26	0.444	0.366	0.284	0.348	0.535	0.715
GLCM_Entropy_log10	2.94	2.98	3.93	2.76	0.204	2.52	2.89	3.57	2.73	2.3	3.27
GLCM_Dissimilarity	6.66	6.96	31	4.91	2.26	3.85	6.77	21.4	43.3	3.09	8.31
GLRLM_SRE	0.946	0.939	0.966	0.927	0.444	0.932	0.949	0.983	0.891	0.894	0.954
GLRLM_LRE	1.33	1.39	1.63	1.43	257	1.35	1.28	1.08	5.38	1.79	1.28
GLRLM_LGRE	0.0000564	0.000038	0.0000105	0.0000438	7.21E-06	0.0000636	0.0000503	0.0000275	0.0000102	0.0000569	0.000045
GLRLM_HGRE	18000	27200	106000	23100	148000	15900	20100	38400	117000	17700	23100
GLRLM_SRLGE	0.0000533	0.0000358	0.0000102	0.0000406	3.75E-06	0.0000592	0.0000477	0.000027	9.45E-06	0.0000508	0.0000429
GLRLM_SRHGE	17100	25500	101000	21400	59100	14800	19200	37800	99500	15800	22100
GLRLM_LRLGE	0.000076	0.0000521	0.0000146	0.0000625	0.0016	0.0000859	0.000065	0.00003	0.0000375	0.000102	0.000058
GLRLM_LRHGE	23800	38100	205000	33200	41300000	21300	25600	41400	820000	31600	29500
GLRLM_GLNU	1040	704	461	1930	4680	631	1840	118	1690	2290	596
GLRLM_RLNU	28500	21400	35300	43000	1370	10100	47300	8430	15300	28100	27700
GLRLM_RP	0.924	0.915	0.928	0.902	0.204	0.91	0.93	0.976	0.731	0.861	0.934
NGLDM_Coarseness	0.000231	0.000373	0.0000476	0.000157	0.0000298	0.000674	0.000118	0.000665	0.0000061	0.000196	0.000322
NGLDM_Contrast	0.168	0.134	1.93	0.114	0.026	0.0553	0.104	1.02	3.02	0.0597	0.318
NGLDM_Busyness	2.53	0.249	0.42	2.33	0.388	0.862	3.79	0.189	1.51	6.63	0.97
GLZLM_SZE	0.605	0.653	0.887	0.563	0.963	0.626	0.629	0.816	0.94	0.438	0.658
GLZLM_LZE	32.1	67	620	257	791000	120	57	2.73	11800	1850	21.6
GLZLM_LGZE	0.0000549	0.00004	0.0000108	0.0000448	9.44E-06	0.0000629	0.0000491	0.0000271	0.0000115	0.0000565	0.0000442
GLZLM_HGZE	18500	26300	102000	22700	121000	16100	20700	38800	102000	17800	23600
GLZLM_SZLGE	0.0000328	0.0000267	9.55E-06	0.0000255	9.13E-06	0.0000391	0.0000306	0.000022	0.0000108	0.0000247	0.0000288
GLZLM_SZHGE	11400	16900	90700	12600	116000	10200	13200	31800	95800	7820	15600
GLZLM_LZLGE	0.00195	0.00239	0.00387	0.0111	4.92	0.00751	0.00324	0.0000804	0.0736	0.106	0.000993
GLZLM_LZHGE	539000	1890000	99700000	5980000	1.27E+11	1920000	1010000	100000	1.9E+09	32300000	487000
GLZLM_GLNU	386	182	187	461	13.4	163	760	89.7	72.4	267	255
GLZLM_ZLNU	4240	3690	22600	4450	1350	1430	8430	4080	11600	952	5570
GLZLM_ZP	0.357	0.348	0.735	0.265	0.0416	0.301	0.399	0.73	0.495	0.123	0.421

Patient	23	24	25	26	27	28	29	30	31	32	33
Type(GBM=2, AO=1)	2	2	2	2	2	2	2	2	2	2	2
minValue	0	119	90	149	0	0	0	386	0	120	0
meanValue	555	316	245	288	252	180	146	2220	203	222	67.7
stdValue	125	113	54.7	41.5	82	60.8	81.3	668	64.6	29.2	90.1
maxValue	1090	1050	415	445	870	655	396	6460	457	318	514
HISTO_Skewness	0.367	1.3	-0.0163	1.2	1.54	1.7	0.0482	1.17	-0.185	-0.0728	1.96
HISTO_Kurtosis	4.67	4.58	2.77	4.81	6.61	9.48	2.47	5.01	2.9	2.77	6.66
HISTO_Entropy_log10	1.67	1.54	1.35	1.16	1.42	1.3	1.48	2.11	1.42	1.08	1.18
HISTO_Energy	0.0281	0.0398	0.0515	0.0926	0.0529	0.0709	0.0367	0.0183	0.0441	0.0954	0.132
SHAPE_Volume (mL)	28.6	71	14.8	3.28	107	43.6	20.2	32.5	88.8	22.5	46.3
GLCM_Homogeneity	0.285	0.328	0.334	0.42	0.429	0.425	0.337	0.0974	0.391	0.474	0.475
GLCM_Energy	0.00174	0.00534	0.00491	0.0128	0.00785	0.0123	0.00401	0.00175	0.0046	0.014	0.0432
GLCM_Contrast	107	111	44.9	10.1	29	41	65.7	3850	28.6	6.22	61.1
GLCM_Correlation	0.661	0.575	0.238	0.666	0.74	0.369	0.455	0.2	0.619	0.615	0.465
GLCM_Entropy_log10	3.08	2.85	2.5	2.13	2.49	2.31	2.64	3.76	2.56	1.98	1.98
GLCM_Dissimilarity	6.86	6.89	4.88	2.4	3.19	3.82	5.68	45.3	3.56	1.88	4.12
GLRLM_SRE	0.95	0.922	0.925	0.906	0.884	0.883	0.919	0.985	0.907	0.894	0.861
GLRLM_LRE	1.29	1.81	1.53	1.48	1.82	2.38	1.79	1.21	1.56	1.61	2.08
GLRLM_LGRE	0.0000419	0.0000576	0.0000643	0.0000599	0.0000635	0.0000712	0.0000762	0.000011	0.000069	0.0000664	0.0000866
GLRLM_HGRE	24500	17900	15700	16800	16000	14200	13400	101000	14700	15100	11900
GLRLM_SRLGE	0.0000398	0.0000527	0.0000595	0.0000542	0.0000558	0.0000626	0.0000698	0.0000109	0.0000625	0.0000593	0.0000735
GLRLM_SRHGE	23300	16600	14500	15200	14200	12600	12300	99000	13300	13500	10400
GLRLM_LRLGE	0.000054	0.00011	0.000098	0.0000888	0.000118	0.000174	0.000139	0.0000125	0.000108	0.000107	0.00019
GLRLM_LRHGE	31500	30700	23900	24700	28600	32800	23500	133000	22800	24200	23300
GLRLM_GLNU	592	1590	549	291	7160	1720	465	119	908	309	1050
GLRLM_RLNU	19100	37800	9260	2590	111000	20500	10900	8030	16800	2540	7690
GLRLM_RP	0.93	0.877	0.898	0.875	0.839	0.828	0.881	0.968	0.876	0.863	0.796
NGLDM_Coarseness	0.000446	0.000128	0.000597	0.00222	0.0000647	0.000198	0.000488	0.000131	0.000431	0.00247	0.000304
NGLDM_Contrast	0.117	0.168	0.188	0.0601	0.0411	0.0519	0.332	3.36	0.105	0.0564	0.205
NGLDM_Busyness	0.281	3.58	3.12	0.767	4.63	2.89	3.73	0.201	2.04	1.69	12
GLZLM_SZE	0.656	0.672	0.522	0.642	0.608	0.619	0.515	0.919	0.576	0.538	0.73
GLZLM_LZE	25.2	1790	114	1430	27200	3520	334	38	557	569	6420
GLZLM_LGZE	0.0000421	0.0000531	0.0000644	0.0000586	0.0000586	0.000067	0.0000742	0.0000114	0.0000681	0.0000652	0.0000764
GLZLM_HGZE	24600	19400	15700	17200	17500	15200	13700	96700	14900	15400	13400
GLZLM_SZLGE	0.0000277	0.0000351	0.0000336	0.0000378	0.0000351	0.0000409	0.0000381	0.0000104	0.0000392	0.0000351	0.0000552
GLZLM_SZHGE	16200	13200	8170	11000	10900	9580	7080	89200	8590	8260	9900
GLZLM_LZLGE	0.00106	0.119	0.00726	0.0874	1.87	0.265	0.0293	0.000244	0.039	0.0387	0.62
GLZLM_LZHGE	605000	26800000	1790000	23400000	397000000	47000000	3890000	6010000	8010000	8370000	66600000
GLZLM_GLNU	206	413	131	36.1	768	200	100	46.4	169	51.7	122
GLZLM_ZLNU	3670	6870	719	246	9900	1920	713	5600	1410	161	1710
GLZLM_ZP	0.401	0.321	0.231	0.17	0.165	0.172	0.194	0.802	0.192	0.156	0.253

Patient	34	35	36	37	38	39	40	41	42	43	44
Type(GBM=2, AO=1)	2	2	2	2	2	2	2	2	2	2	2
minValue	0	43	3	247	0	0	412	754	33	830	0
meanValue	407	325	719	771	4020	288	5230	3830	174	2510	274
stdValue	136	114	352	153	1720	128	1040	1390	73.9	1120	65.7
maxValue	1130	934	1860	1280	9820	1250	9650	8900	822	7230	541
HISTO_Skewness	0.553	0.617	0.64	-0.0811	1.19	1.58	0.129	0.596	2.22	1.66	0.21
HISTO_Kurtosis	4.67	2.99	3.18	2.3	3.9	5.99	2.88	3.18	11.4	5.35	3.7
HISTO_Entropy_log10	1.69	1.62	2.11	1.78	0.903	1.59	0.0252	0.889	1.33	1.91	1.43
HISTO_Energy	0.0276	0.027	0.00963	0.018	0.48	0.0365	0.988	0.512	0.0679	0.0556	0.0446
SHAPE_Volume (mL)	51.5	64.6	11.3	64.4	31.6	11.3	65	52.5	9.09	21.6	21.4
GLCM_Homogeneity	0.259	0.282	0.143	0.259	0.482	0.359	0.991	0.622	0.348	0.192	0.309
GLCM_Energy	0.00179	0.0022	0.000221	0.00104	0.221	0.0047	0.981	0.356	0.00826	0.0157	0.00322
GLCM_Contrast	188	126	762	148	1650	114	27.7	1530	76.1	3250	51.2
GLCM_Correlation	0.445	0.557	0.696	0.676	0.29	0.574	0.178	0.604	0.385	0.428	0.421
GLCM_Entropy_log10	3.14	3.01	3.83	3.24	1.93	2.81	0.0414	1.68	2.47	3.3	2.66
GLCM_Dissimilarity	9.11	7.87	19.2	8.53	24.5	6.21	0.327	18.5	5.54	37.3	5.07
GLRLM_SRE	0.951	0.941	0.982	0.945	0.819	0.916	0.179	0.77	0.923	0.947	0.945
GLRLM_LRE	1.29	1.49	1.08	1.37	11.3	1.6	389	17.4	1.7	1.83	1.29
GLRLM_LGRE	0.0000513	0.0000571	0.0000378	0.0000323	7.62E-06	0.0000607	6.39E-06	8.42E-06	0.0000723	0.0000109	0.0000615
GLRLM_HGRE	20200	18000	31100	31900	139000	17100	159000	130000	14100	101000	16400
GLRLM_SRLGE	0.0000487	0.0000535	0.000037	0.0000305	6.48E-06	0.0000552	1.29E-06	6.97E-06	0.0000665	0.0000106	0.0000581
GLRLM_SRHGE	19300	17000	30600	30100	110000	15800	27000	93500	13000	92900	15500
GLRLM_LRLGE	0.0000671	0.000089	0.0000411	0.0000446	0.0000715	0.0001	0.00242	0.00011	0.000125	0.0000163	0.0000793
GLRLM_LRHGE	25700	25900	33200	43200	1790000	26400	62600000	2770000	23400	232000	21100
GLRLM_GLNU	924	1130	111	736	1780	539	3200	1600	375	106	218
GLRLM_RLNU	31000	37400	11100	36300	3760	13300	532	3970	5060	3400	4330
GLRLM_RP	0.931	0.909	0.975	0.922	0.642	0.876	0.171	0.513	0.89	0.89	0.927
NGLDM_Coarseness	0.000197	0.000148	0.00096	0.000213	0.0000177	0.000448	0.000342	0.0000187	0.000985	0.00011	0.00149
NGLDM_Contrast	0.194	0.202	0.807	0.293	0.979	0.169	0.000816	1.32	0.0792	4.37	0.1
NGLDM_Busyness	0.851	2.36	0.0909	0.84	0.969	0.678	0.376	0.976	0.765	0.301	0.366
GLZLM_SZE	0.715	0.639	0.832	0.638	0.926	0.723	0.978	0.916	0.587	0.897	0.636
GLZLM_LZE	56.6	361	3	39.2	16000	782	3470000	28200	172	327	29
GLZLM_LGZE	0.0000497	0.0000544	0.0000362	0.0000316	8.89E-06	0.0000556	8.75E-06	9.91E-06	0.0000671	0.0000116	0.0000615
GLZLM_HGZE	21100	18800	32400	32600	119000	18900	133000	110000	15300	92200	16500
GLZLM_SZLGE	0.0000352	0.0000344	0.0000295	0.00002	8.25E-06	0.0000393	8.59E-06	9.05E-06	0.0000383	0.0000104	0.0000391
GLZLM_SZHGE	15300	12100	27500	21000	110000	14000	130000	101000	9210	83200	10500
GLZLM_LZLGE	0.00317	0.0256	0.000126	0.00135	0.0993	0.0536	21.6	0.176	0.0131	0.00204	0.00178
GLZLM_LZHGE	1020000	5120000	83000	1170000	2.57E+09	11400000	5.57E+11	4.54E+09	2270000	52400000	476000
GLZLM_GLNU	332	469	77.4	290	25.8	125	2.9	23.5	51.7	21.4	81.5
GLZLM_ZLNU	7990	6660	5630	6020	2140	2830	109	2900	489	2190	766
GLZLM_ZP	0.453	0.374	0.729	0.36	0.278	0.315	0.00573	0.255	0.232	0.659	0.384

Patient	45	46	47	48	49	50	51	52	53	54	55
Type(GBM=2, AO=1)	2	2	2	2	2	2	2	2	2	2	2
minValue	0	452	2	0	207	0	2900	34	167	229	273
meanValue	95.7	1290	303	65.9	356	182	4110	207	437	486	895
stdValue	88	381	162	59.9	52.9	95.9	410	125	127	176	235
maxValue	706	4450	1150	326	545	890	5090	1030	872	1200	1780
HISTO_Skewness	1.82	2.14	1.23	1.25	0.139	1.08	-0.116	1.78	0.682	0.705	0.485
HISTO_Kurtosis	8.38	12	4.29	3.87	3.14	5.72	2.19	7.35	3.29	2.72	3.12
HISTO_Entropy_log10	1.34	2.09	1.7	1.19	1.33	1.56	0.00321	1.53	1.69	1.76	1.97
HISTO_Energy	0.0762	0.00985	0.0303	0.101	0.0564	0.0324	0.998	0.0439	0.0241	0.0214	0.0124
SHAPE_Volume (mL)	36.7	19.9	79	12.3	4.57	183	3.89	8.57	4.35	42.2	10.6
GLCM_Homogeneity	0.481	0.101	0.302	0.474	0.356	0.366	0.998	0.331	0.212	0.241	0.133
GLCM_Energy	0.0267	0.000237	0.00357	0.0318	0.00597	0.00334	0.995	0.0055	0.00125	0.00167	0.000349
GLCM_Contrast	53.9	1500	255	18.3	26.4	71.8	0.257	82.6	198	311	740
GLCM_Correlation	0.569	0.323	0.511	0.577	0.423	0.54	-0.00129	0.738	0.329	0.487	0.308
GLCM_Entropy_log10	2.23	3.7	3.06	1.97	2.4	2.79	0.00849	2.75	3.05	3.23	3.56
GLCM_Dissimilarity	3.86	26.7	9.68	2.74	3.79	5.12	0.0257	5.61	10.2	12	20.1
GLRLM_SRE	0.848	0.991	0.935	0.878	0.937	0.905	0.325	0.932	0.97	0.952	0.986
GLRLM_LRE	4.01	1.04	1.38	1.91	1.41	1.87	74.2	1.36	1.15	1.31	1.06
GLRLM_LGRE	0.0000822	0.0000202	0.0000601	0.0000867	0.0000542	0.0000718	6.22E-06	0.0000692	0.000049	0.0000462	0.0000289
GLRLM_HGRE	12500	53900	17600	11700	18600	14300	161000	15000	21000	22800	36700
GLRLM_SRLGE	0.0000689	0.00002	0.0000557	0.0000755	0.0000508	0.0000647	2.03E-06	0.000064	0.0000475	0.0000436	0.0000285
GLRLM_SRHGE	10700	53400	16600	10400	17400	13000	52300	14100	20400	21900	36200
GLRLM_LRLGE	0.000353	0.000021	0.0000855	0.000172	0.0000766	0.000137	0.000461	0.0000965	0.0000569	0.0000629	0.0000309
GLRLM_LRHGE	46500	56300	23300	21500	26200	26200	11900000	19800	24100	28600	38900
GLRLM_GLNU	1140	60.2	663	193	170	3660	391	268	74.4	570	89
GLRLM_RLNU	15100	6020	20900	1810	2670	94300	129	5770	2890	25200	6940
GLRLM_RP	0.765	0.987	0.908	0.823	0.912	0.863	0.364	0.905	0.959	0.929	0.981
NGLDM_Coarseness	0.00026	0.00109	0.000246	0.00242	0.00253	0.0000711	0.0761	0.00126	0.00224	0.000185	0.00102
NGLDM_Contrast	0.0899	0.926	0.338	0.137	0.112	0.0938	0.0000198	0.145	0.418	0.652	0.672
NGLDM_Busyness	4.57	0.0484	1.12	2.2	0.636	6.42	3.29	0.451	0.251	2.58	0.115
GLZLM_SZE	0.487	0.895	0.747	0.71	0.58	0.559	0.5	0.721	0.737	0.737	0.843
GLZLM_LZE	14700	1.68	724	934	46	1940	582000	423	6.57	176	2.21
GLZLM_LGZE	0.0000759	0.0000202	0.0000546	0.0000802	0.0000543	0.0000677	6.38E-06	0.0000636	0.000048	0.000043	0.0000286
GLZLM_HGZE	13600	53900	19500	12600	18500	15300	157000	16400	21500	24400	37100
GLZLM_SZLGE	0.0000359	0.000018	0.00004	0.0000564	0.0000314	0.0000373	3.27E-06	0.0000452	0.000035	0.0000311	0.000024
GLZLM_SZHGE	6890	48500	14800	9060	10800	8700	76400	12000	16000	18300	31400
GLZLM_LZLGE	1.38	0.0000332	0.0508	0.0885	0.00248	0.152	3.62	0.0339	0.000339	0.0105	0.000066
GLZLM_LZHGE	158000000	96500	10400000	9870000	855000	25000000	9.36E+10	5270000	130000	2960000	78600
GLZLM_GLNU	98.7	50.5	193	35.6	47.4	614	1	74	41.9	265	69
GLZLM_ZLNU	579	4040	5920	353	317	6630	1	1390	947	7700	3780
GLZLM_ZP	0.0958	0.852	0.424	0.256	0.301	0.17	0.00185	0.381	0.581	0.503	0.775

Patient	56	57	58	59	60	61	62	63	64	65	66
Type(GBM=2, AO=1)	2	2	2	2	2	2	2	2	2	2	2
minValue	179	131	486	126	538	0	0	551	36	584	67
meanValue	558	926	1890	471	1490	380	477	2130	646	2360	247
stdValue	82.3	276	449	144	650	155	162	452	294	856	35.7
maxValue	830	2080	6960	1280	4930	835	1270	5830	2060	5150	468
HISTO_Skewness	0.038	0.361	1.81	0.868	2.29	0.219	-0.458	1.77	0.383	0.44	0.834
HISTO_Kurtosis	2.7	2.76	9.52	3.84	8.98	2.55	2.72	10.3	2.4	2.47	5.97
HISTO_Entropy_log1	1.53	2.04	2.12	1.73	2.12	1.78	1.8	2.11	1.99	1.98	1.13
HISTO_Energy	0.0336	0.0103	0.00904	0.0227	0.0106	0.0189	0.0181	0.00984	0.0121	0.0617	0.0989
SHAPE_Volume (mL)	26.2	23.2	119	28.7	14	46.3	61.1	56	37.2	15.2	92.6
GLCM_Homogeneity	0.33	0.133	0.141	0.274	0.104	0.254	0.239	0.107	0.18	0.142	0.439
GLCM_Energy	0.00295	0.000255	0.000313	0.00168	0.00058	0.000964	0.000858	0.000295	0.000649	0.0106	0.0141
GLCM_Contrast	45.5	944	1790	125	3430	151	208	1450	795	4800	12.8
GLCM_Correlation	0.607	0.316	0.463	0.638	0.418	0.681	0.559	0.448	0.552	0.444	0.388
GLCM_Entropy_log10	2.74	3.74	4	3.06	3.56	3.29	3.33	3.92	3.62	3.28	2.1
GLCM_Dissimilarity	4.88	22.9	28.2	7.42	39.3	8.6	9.82	27.6	19.3	50.6	2.47
GLRLM_SRE	0.922	0.983	0.978	0.951	0.986	0.952	0.956	0.988	0.971	0.964	0.891
GLRLM_LRE	1.57	1.08	1.15	1.25	1.09	1.3	1.27	1.08	1.17	1.44	1.62
GLRLM_LGRE	0.0000413	0.0000285	0.0000127	0.0000468	0.0000184	0.0000538	0.0000474	0.0000108	0.0000398	0.0000113	0.0000638
GLRLM_HGRE	24500	38100	84600	22200	62600	19500	22200	98000	28400	105000	15700
GLRLM_SRLGE	0.0000381	0.000028	0.0000124	0.0000443	0.0000182	0.0000511	0.0000454	0.0000107	0.0000384	0.0000111	0.0000568
GLRLM_SRHGE	22600	37500	82700	21200	61300	18600	21200	96700	27700	99800	14000
GLRLM_LRLGE	0.0000646	0.0000309	0.0000143	0.0000595	0.0000195	0.0000714	0.00006	0.0000115	0.0000483	0.0000142	0.000103
GLRLM_LRHGE	38500	41000	100000	27100	73300	25000	28400	109000	32200	175000	25300
GLRLM_GLNU	539	159	1090	204	41.6	1320	1680	159	291	186	2580
GLRLM_RLNU	13700	14900	117000	8210	4070	62600	84000	16200	23200	4040	21000
GLRLM_RP	0.893	0.976	0.967	0.934	0.978	0.93	0.936	0.981	0.958	0.931	0.856
NGLDM_Coarseness	0.00062	0.000432	0.0000418	0.00109	0.000712	0.000128	0.0000872	0.000286	0.000278	0.0000976	0.000246
NGLDM_Contrast	0.106	0.768	1.26	0.211	2.83	0.409	0.262	1.14	0.678	5.82	0.0291
NGLDM_Busyness	0.621	0.179	0.795	0.248	0.0745	2.92	1.49	0.108	0.337	0.271	3.76
GLZLM_SZE	0.532	0.816	0.82	0.738	0.899	0.693	0.685	0.886	0.802	0.938	0.614
GLZLM_LZE	107	2.63	16.6	48.1	8.66	45.4	22.4	4.44	13.5	232	11600
GLZLM_LGZE	0.0000417	0.0000281	0.0000124	0.000044	0.0000185	0.0000523	0.0000486	0.0000109	0.0000367	0.0000123	0.0000626
GLZLM_HGZE	24300	38600	85700	23600	60500	20000	21700	96700	30300	94000	16100
GLZLM_SZLGE	0.0000222	0.0000228	0.00001	0.0000319	0.0000165	0.0000363	0.0000336	9.65E-06	0.0000289	0.0000114	0.0000384
GLZLM_SZHGE	12900	31700	71400	17800	55200	13900	14700	86000	24700	88800	9920
GLZLM_LZLGE	0.00433	0.0000771	0.000129	0.0026	0.0000749	0.00296	0.00102	0.0000357	0.000733	0.00145	0.747
GLZLM_LZHGE	2660000	97000	2380000	898000	1230000	737000	518000	604000	263000	37200000	180000000
GLZLM_GLNU	132	117	701	91.5	30.3	591	754	120	163	17.3	263
GLZLM_ZLNU	1120	7230	7240	2380	2720	14300	18900	10400	9720	2820	1780
GLZLM_ZP	0.233	0.732	0.695	0.476	0.821	0.429	0.438	0.814	0.625	0.7	0.157

Patient	67	68	69	70	71	72	73	74	75	76	77
Type(GBM=2, AO=1)	2	2	2	2	2	2	2	2	2	2	1
minValue	49	92	111	4	0	21	179	127	140	91	2340
meanValue	564	1240	269	351	198	316	610	302	358	2830	3010
stdValue	122	543	43	127	58.3	139	161	72.1	121	754	292
maxValue	973	3930	410	1090	676	1370	1060	556	935	5420	5300
HISTO_Skewness	-0.164	1.15	0.158	0.362	1.82	1.26	-0.207	0.0657	1.14	-0.832	2.13
HISTO_Kurtosis	3.36	5.01	3.1	3.2	12.1	6.01	2.82	2.61	4.1	4.14	13.1
HISTO_Entropy_log10	1.69	2.25	1.24	1.71	1.32	1.69	1.81	1.47	1.62	1.51	1.34
HISTO_Energy	0.0253	0.00789	0.0664	0.0228	0.0624	0.0249	0.0181	0.0383	0.0305	0.206	0.0565
SHAPE_Volume (mL)	10	50	6.75	97.5	32.9	132	25.2	33.4	33	28.8	27.5
GLCM_Homogeneity	0.273	0.112	0.391	0.246	0.397	0.251	0.203	0.32	0.282	0.38	0.301
GLCM_Energy	0.00171	0.000207	0.00889	0.00121	0.0082	0.00159	0.000716	0.00278	0.00282	0.108	0.00444
GLCM_Contrast	118	3760	20	185	47.2	230	225	48.7	149	2980	52.6
GLCM_Correlation	0.551	0.207	0.405	0.412	0.312	0.412	0.507	0.562	0.511	0.414	0.279
GLCM_Entropy_log10	3.07	3.91	2.25	3.2	2.42	3.17	3.32	2.75	2.97	2.45	2.49
GLCM_Dissimilarity	7.59	39.8	3.31	9.56	4.14	9.94	10.6	4.99	8.21	29.9	5.02
GLRLM_SRE	0.946	0.987	0.905	0.952	0.895	0.953	0.971	0.937	0.943	0.893	0.957
GLRLM_LRE	1.35	1.06	1.84	1.34	2.13	1.32	1.13	1.4	1.49	5.19	1.2
GLRLM_LGRE	0.0000414	0.0000231	0.0000617	0.0000555	0.0000693	0.0000584	0.0000396	0.0000589	0.0000544	9.64E-06	0.00983
GLRLM_HGRE	24800	53100	16300	18700	14600	17800	26300	17200	18900	127000	268
GLRLM_SRLGE	0.0000391	0.0000228	0.0000559	0.0000527	0.0000619	0.0000553	0.0000384	0.0000551	0.000051	8.95E-06	0.00945
GLRLM_SRHGE	23500	52400	14700	17800	13100	17100	25600	16100	17900	110000	259
GLRLM_LRLGE	0.0000561	0.0000244	0.000114	0.000076	0.000149	0.0000798	0.0000447	0.0000831	0.0000838	0.0000358	0.0115
GLRLM_LRHGE	33400	56900	29900	24400	30700	22600	29800	23900	27000	800000	310
GLRLM_GLNU	372	114	287	1490	1220	2200	152	819	641	487	82.9
GLRLM_RLNU	13300	14200	3670	58800	16600	80500	7830	18500	19500	5010	1340
GLRLM_RP	0.923	0.982	0.869	0.929	0.851	0.93	0.962	0.913	0.912	0.736	0.943
NGLDM_Coarseness	0.000581	0.000415	0.00144	0.000102	0.000311	0.0000726	0.00105	0.000364	0.000276	0.0000356	0.00403
NGLDM_Contrast	0.183	2.09	0.0908	0.224	0.0438	0.156	0.478	0.193	0.29	2.06	0.13
NGLDM_Busyness	0.287	0.0745	1.2	2.24	1.54	2.58	0.244	3.41	2.19	0.238	0.288
GLZLM_SZE	0.646	0.878	0.468	0.658	0.563	0.716	0.745	0.571	0.698	0.919	0.68
GLZLM_LZE	46.8	2.46	311	52.6	1030	436	5.17	65.7	199	3410	9.99
GLZLM_LGZE	0.0000411	0.000023	0.0000619	0.0000532	0.0000661	0.0000551	0.0000398	0.0000578	0.000051	0.0000107	0.0105
GLZLM_HGZE	25100	53800	16300	19500	15400	18900	26300	17500	20200	116000	315
GLZLM_SZLGE	0.0000265	0.0000199	0.0000291	0.0000346	0.0000363	0.0000387	0.0000297	0.0000329	0.000035	9.89E-06	0.00624
GLZLM_SZHGE	16200	48100	7570	13000	8950	13900	19500	10100	14300	106000	239
GLZLM_LZLGE	0.00191	0.0000505	0.0191	0.00348	0.0712	0.033	0.000203	0.0042	0.0126	0.0212	0.08
GLZLM_LZHGE	1160000	172000	5070000	810000	14800000	5770000	137000	1040000	3150000	549000000	2140
GLZLM_GLNU	126	80.4	37.4	643	147	1070	90.4	275	241	35.4	34.7
GLZLM_ZLNU	2300	8780	148	11700	1110	22500	2720	2150	4500	3570	321
GLZLM_ZP	0.367	0.805	0.138	0.413	0.157	0.488	0.607	0.301	0.413	0.496	0.476

Patient	78	79	80	81	82	83	84	85	86	87	88
Type(GBM=2, AO=1)	1	1	1	1	1	1	1	1	1	1	1
minValue	2500	1200	387	398	57	193	16.2	987	514	1480	2910
meanValue	5830	2450	573	443	190	315	271	2010	1200	1830	3720
stdValue	1070	434	45.2	21.7	63.3	47.4	145	586	227	197	280
maxValue	7770	4320	779	529	394	454	709	3330	1830	2870	4560
HISTO_Skewness	-0.398	0.0913	0.035	0.928	0.146	-0.151	0.666	0.345	-0.735	2.65	0.351
HISTO_Kurtosis	2.39	3.76	4.77	3.82	2.3	2.75	2.79	1.79	3.29	11.7	3.49
HISTO_Entropy_log10	1.66	1.55	1.46	1.59	1.66	1.66	1.69	1.71	1.62	1.36	1.57
HISTO_Energy	0.0244	0.034	0.0422	0.0312	0.0239	0.0252	0.0236	0.0217	0.0286	0.06	0.0339
SHAPE_Volume (mL)	7.81	6.37	2.07	2.99	1.29	2.33	0.886	5.48	10.7	10.2	1.27
GLCM_Homogeneity	0.247	0.256	0.311	0.207	0.209	0.194	0.21	0.207	0.254	0.292	0.163
GLCM_Energy	0.00191	0.00189	0.00373	0.00233	0.00134	0.00177	0.00177	0.00198	0.00159	0.00677	0.00638
GLCM_Contrast	84.3	75.6	50.2	142	110	135	111	196	109	81.5	192
GLCM_Correlation	0.767	0.564	0.498	0.266	0.617	0.475	0.623	0.479	0.529	0.145	0.15
GLCM_Entropy_log10	2.82	2.84	2.6	2.72	2.96	2.83	2.82	2.8	3.01	2.33	2.24
GLCM_Dissimilarity	6.81	6.38	5.17	8.99	8.11	9.09	8.09	10.6	7.5	5.7	11.1
GLRLM_SRE	0.967	0.964	0.944	0.977	0.977	0.981	0.978	0.969	0.96	0.957	0.988
GLRLM_LRE	1.15	1.16	1.33	1.11	1.1	1.09	1.09	1.17	1.22	1.22	1.05
GLRLM_LGRE	0.00437	0.0042	0.00239	0.00704	0.00632	0.0045	0.0122	0.00516	0.00349	0.00754	0.00661
GLRLM_HGRE	1830	770	1010	623	817	1060	762	1070	1290	366	1120
GLRLM_SRLGE	0.00415	0.00408	0.00228	0.00675	0.00611	0.00444	0.0117	0.00498	0.0033	0.00725	0.0066
GLRLM_SRHGE	1760	745	953	612	802	1040	750	1040	1240	357	1110
GLRLM_LRLGE	0.00527	0.00475	0.00295	0.00828	0.00721	0.00483	0.0142	0.00612	0.00467	0.00899	0.00666
GLRLM_LRHGE	2150	883	1340	671	880	1150	812	1210	1570	413	1170
GLRLM_GLNU	25.1	62.7	101	30.3	31.9	27.5	19	27.4	289	39.8	6.63
GLRLM_RLNU	952	1700	2140	925	1260	1040	764	1190	9240	615	192
GLRLM_RP	0.957	0.953	0.924	0.968	0.969	0.974	0.97	0.957	0.945	0.942	0.985
NGLDM_Coarseness	0.0103	0.00493	0.00325	0.00652	0.00694	0.00737	0.0122	0.00724	0.000778	0.00816	0.028
NGLDM_Contrast	0.368	0.162	0.102	0.357	0.434	0.39	0.479	0.736	0.298	0.261	0.707
NGLDM_Busyness	0.0395	0.107	0.132	0.125	0.123	0.0738	0.0896	0.128	0.508	0.128	0.0254
GLZLM_SZE	0.685	0.69	0.619	0.774	0.772	0.794	0.771	0.701	0.658	0.675	0.876
GLZLM_LZE	5.58	6.69	25.9	3.59	3.68	2.85	3.51	5.51	10.4	11.9	1.75
GLZLM_LGZE	0.00343	0.00454	0.0028	0.00567	0.00522	0.00496	0.00977	0.00513	0.00283	0.0087	0.00766
GLZLM_HGZE	1690	817	1030	695	876	1060	855	1180	1290	503	1140
GLZLM_SZLGE	0.0012	0.00299	0.00138	0.00343	0.00309	0.0043	0.00618	0.00371	0.00165	0.00684	0.00751
GLZLM_SZHGE	1120	587	658	574	697	849	709	867	845	406	1000
GLZLM_LZLGE	0.0217	0.0216	0.0362	0.0296	0.0298	0.0113	0.0525	0.0318	0.0482	0.082	0.00852
GLZLM_LZHGE	12100	4660	25700	1720	2360	2920	2100	4620	13100	2540	1920
GLZLM_GLNU	14.3	32.7	34.5	18.9	21.7	19.4	12	14.6	137	14	4.82
GLZLM_ZLNU	263	463	346	374	507	463	306	341	2050	143	120
GLZLM_ZP	0.557	0.538	0.37	0.664	0.664	0.707	0.663	0.561	0.474	0.466	0.826

Patient	89	90	91	92	93	94	95	96	97	98	99
Type(GBM=2, AO=1)	1	1	1	1	1	1	1	1	1	1	1
minValue	97.3	316	2290	173	229	165	198	348	160	103	341
meanValue	288	600	3130	636	648	283	313	802	452	246	550
stdValue	52.1	181	543	142	171	57	69.6	179	102	59	89.5
maxValue	523	1170	5310	1010	1590	524	526	1330	754	574	899
HISTO_Skewness	-0.579	0.51	1.46	-0.347	1.49	0.383	0.134	0.0966	0.183	1.31	0.505
HISTO_Kurtosis	3.57	2.53	5.09	3.06	6.88	2.93	2.13	3.21	2.93	6.92	3.26
HISTO_Entropy_log10	1.49	1.69	1.55	1.63	1.45	1.6	1.66	1.64	1.62	1.47	1.61
HISTO_Energy	0.0392	0.0224	0.0364	0.0269	0.0459	0.028	0.0249	0.0282	0.0281	0.0423	0.0289
SHAPE_Volume (mL)	4.37	9.65	8.47	1.47	10.6	4.33	2	8.93	0.382	4.06	15.8
GLCM_Homogeneity	0.329	0.245	0.258	0.233	0.249	0.214	0.27	0.241	0.207	0.262	0.269
GLCM_Energy	0.00297	0.00181	0.00369	0.00148	0.00306	0.00197	0.00285	0.00166	0.00288	0.00302	0.00167
GLCM_Contrast	27.9	177	127	89.9	92.7	122	104	160	106	102	81.5
GLCM_Correlation	0.769	0.522	0.527	0.611	0.245	0.427	0.495	0.451	0.473	0.262	0.527
GLCM_Entropy_log10	2.69	3.04	2.54	2.93	2.68	2.85	2.78	2.99	2.59	2.69	2.95
GLCM_Dissimilarity	3.98	9.79	7.54	7.13	7.05	8.71	7.49	9.18	8.04	7.14	6.41
GLRLM_SRE	0.945	0.95	0.966	0.972	0.964	0.966	0.95	0.959	0.979	0.956	0.957
GLRLM_LRE	1.29	1.49	1.16	1.12	1.17	1.22	1.67	1.23	1.09	1.24	1.22
GLRLM_LGRE	0.00249	0.0166	0.0145	0.004	0.0047	0.00734	0.0184	0.00821	0.00398	0.00579	0.00512
GLRLM_HGRE	909	685	475	1410	482	577	731	1040	1150	465	711
GLRLM_SRLGE	0.00238	0.0153	0.0141	0.00392	0.00456	0.00701	0.0169	0.00756	0.00394	0.00557	0.00487
GLRLM_SRHGE	858	660	466	1370	469	560	701	1000	1130	448	687
GLRLM_LRLGE	0.00305	0.0378	0.0163	0.0043	0.00542	0.00986	0.0465	0.0131	0.00411	0.00694	0.00647
GLRLM_LRHGE	1180	863	520	1590	539	671	1020	1240	1230	557	829
GLRLM_GLNU	153	209	19	41.1	61.5	137	89.2	94.8	12.4	77	220
GLRLM_RLNU	3430	8570	489	1430	1250	4570	3330	3080	421	1670	6890
GLRLM_RP	0.925	0.924	0.955	0.963	0.951	0.951	0.926	0.943	0.972	0.94	0.942
NGLDM_Coarseness	0.00326	0.000987	0.0152	0.00634	0.00429	0.0019	0.00427	0.00258	0.0178	0.00323	0.00118
NGLDM_Contrast	0.0944	0.439	0.391	0.255	0.189	0.213	0.28	0.337	0.392	0.189	0.233
NGLDM_Busyness	0.163	1.05	0.0834	0.0657	0.177	0.39	0.18	0.207	0.035	0.233	0.563
GLZLM_SZE	0.596	0.612	0.717	0.754	0.728	0.677	0.624	0.673	0.806	0.667	0.68
GLZLM_LZE	26.5	35.9	6.75	4.47	8.74	9.41	34.3	10.9	3	12.9	12.3
GLZLM_LGZE	0.0031	0.00579	0.0159	0.00451	0.00534	0.00573	0.0052	0.00379	0.00488	0.00645	0.00469
GLZLM_HGZE	898	889	599	1390	574	649	928	1120	1180	543	816
GLZLM_SZLGE	0.00175	0.00203	0.0112	0.0033	0.00431	0.00349	0.00144	0.00152	0.00455	0.00407	0.0029
GLZLM_SZHGE	533	595	490	1030	456	460	633	774	976	401	585
GLZLM_LZLGE	0.0417	2.16	0.0728	0.0115	0.0374	0.114	1.72	0.29	0.00791	0.0566	0.0742
GLZLM_LZHGE	24800	8020	1930	6420	2870	3500	10700	9110	3200	4880	6040
GLZLM_GLNU	50.4	82.2	8.6	24.9	27.4	74.7	35.8	44.4	8.26	30.3	103
GLZLM_ZLNU	489	1320	147	521	378	1120	517	726	195	362	1660
GLZLM_ZP	0.348	0.371	0.554	0.619	0.536	0.51	0.364	0.481	0.707	0.447	0.477

Patient	100	101	102	103	104	105	106	107	108	109	110
Type(GBM=2, AO=1)	1	1	1	1	1	1	1	1	1	1	1
minValue	206	176	193	1350	905	192	175	197	179	3220	1160
meanValue	448	826	244	1790	1780	251	326	324	307	4590	2040
stdValue	84.1	195	21.7	172	580	23.7	52.5	51.6	33.8	845	284
maxValue	823	1230	320	2230	3920	339	529	470	415	6520	3380
HISTO_Skewness	0.314	-0.535	0.68	-0.00782	1.16	0.278	-0.317	0.148	-0.282	0.42	0.497
HISTO_Kurtosis	3.43	2.26	2.95	2.52	3.58	3.15	3.16	2.63	3.82	2.38	4.38
HISTO_Entropy_log10	1.55	1.62	1.61	1.68	1.58	1.61	1.56	1.68	1.56	1.69	1.5
HISTO_Energy	0.0339	0.0283	0.0281	0.0235	0.0351	0.0288	0.0325	0.0242	0.0341	0.0235	0.0386
SHAPE_Volume (mL)	3.42	2.21	4.55	3.91	26.4	1.33	0.597	3.25	0.873	1.46	12.2
GLCM_Homogeneity	0.288	0.3	0.231	0.196	0.273	0.256	0.239	0.216	0.364	0.121	0.25
GLCM_Energy	0.00205	0.00216	0.00172	0.00215	0.00288	0.00225	0.00296	0.002	0.00341	0.00683	0.00296
GLCM_Contrast	43.2	61.1	92.2	140	98	80.5	77.8	315	23.2	622	82.6
GLCM_Correlation	0.694	0.762	0.599	0.553	0.648	0.599	0.496	0.0467	0.842	-0.0468	0.317
GLCM_Entropy_log10	2.84	2.86	2.9	2.72	2.81	2.77	2.6	3	2.68	2.21	2.64
GLCM_Dissimilarity	4.98	5.38	7.34	9.16	6.88	6.75	6.71	13.3	3.52	19.5	6.56
GLRLM_SRE	0.957	0.955	0.969	0.979	0.955	0.967	0.973	0.937	0.934	0.987	0.971
GLRLM_LRE	1.19	1.21	1.15	1.09	1.23	1.22	1.12	2.31	1.39	1.06	1.14
GLRLM_LGRE	0.00475	0.00208	0.00294	0.00461	0.00836	0.00815	0.00517	0.00303	0.00174	0.0195	0.00377
GLRLM_HGRE	735	1720	825	1220	531	789	868	1070	1330	1010	744
GLRLM_SRLGE	0.0045	0.00202	0.00285	0.00455	0.00793	0.0076	0.00512	0.00286	0.00165	0.0188	0.00371
GLRLM_SRHGE	706	1630	803	1190	517	765	843	1010	1240	995	725
GLRLM_LRLGE	0.00589	0.00232	0.00335	0.00488	0.0106	0.0125	0.00537	0.00747	0.00222	0.0232	0.00405
GLRLM_LRHGE	862	2130	923	1320	598	947	973	2420	1850	1050	830
GLRLM_GLNU	148	75.2	64.8	15.3	116	48.2	16.9	247	160	4.66	39.4
GLRLM_RLNU	3940	2420	2140	620	3060	1560	488	9560	4070	194	962
GLRLM_RP	0.943	0.939	0.958	0.972	0.939	0.952	0.964	0.921	0.908	0.983	0.961
NGLDM_Coarseness	0.00253	0.00456	0.0035	0.0118	0.0025	0.00575	0.0178	0.000567	0.00298	0.0186	0.00696
NGLDM_Contrast	0.124	0.25	0.301	0.494	0.32	0.225	0.287	0.565	0.0891	1.9	0.207
NGLDM_Busyness	0.234	0.0846	0.217	0.0487	0.444	0.113	0.0383	1.04	0.124	0.046	0.0938
GLZLM_SZE	0.674	0.709	0.704	0.788	0.689	0.696	0.766	0.374	0.586	0.879	0.733
GLZLM_LZE	13	20.2	5.61	3.17	20	8.27	4.06	45.2	51.5	2.1	5.29
GLZLM_LGZE	0.00413	0.00265	0.00308	0.00521	0.00742	0.00485	0.00692	0.00269	0.00233	0.0117	0.00484
GLZLM_HGZE	782	1540	899	1260	696	815	842	1120	1380	1050	806
GLZLM_SZLGE	0.00258	0.00179	0.0023	0.0044	0.00459	0.0025	0.00643	0.000886	0.00128	0.00508	0.00428
GLZLM_SZHGE	539	1060	683	1020	529	578	630	431	823	942	620
GLZLM_LZLGE	0.0556	0.0157	0.0163	0.011	0.206	0.11	0.0117	0.139	0.0517	0.0819	0.0136
GLZLM_LZHGE	8230	43400	3750	3570	4000	6140	3670	43700	66800	1800	3370
GLZLM_GLNU	65.9	32.3	35.3	10.4	42	24.8	10.2	66.5	43.1	3.48	21.3
GLZLM_ZLNU	923	635	628	267	760	415	188	539	520	121	317
GLZLM_ZP	0.469	0.472	0.569	0.686	0.475	0.524	0.636	0.257	0.307	0.809	0.591

Patient	111	112	113	114	115	116	117	118	119	120	121
Type(GBM=2, AO=1)	1	1	1	1	1	1	1	1	1	1	1
minValue	135	2460	178	3080	204	1420	98.2	415	272	331	232
meanValue	190	4480	361	4510	435	1770	137	504	339	725	537
stdValue	32.3	720	64.2	544	116	170	14.8	26	40	130	112
maxValue	340	6920	561	6230	649	2120	193	593	547	1160	1060
HISTO_Skewness	1.59	0.267	0.522	0.297	-0.322	-0.117	0.595	-0.296	1.89	-0.0284	0.201
HISTO_Kurtosis	6.33	3.29	2.58	2.83	1.94	2.07	3.27	2.86	7.94	2.69	3.26
HISTO_Entropy_log10	1.53	1.59	1.61	1.64	1.75	1.73	1.6	1.58	1.46	1.6	1.54
HISTO_Energy	0.0378	0.031	0.0285	0.0266	0.0189	0.0209	0.0296	0.0302	0.0443	0.0284	0.0333
SHAPE_Volume (mL)	0.87	9.32	5.08	3.35	1.69	0.3	3.43	12.7	0.392	1.81	3.01
GLCM_Homogeneity	0.245	0.218	0.423	0.212	0.165	0.116	0.346	0.323	0.303	0.272	0.28
GLCM_Energy	0.00248	0.00234	0.00332	0.00191	0.00314	0.0151	0.00317	0.00199	0.00428	0.00167	0.00201
GLCM_Contrast	121	116	12.7	178	216	898	21.9	25	47.8	66.6	44.5
GLCM_Correlation	0.445	0.394	0.94	0.233	0.191	-0.209	0.828	0.85	0.706	0.672	0.669
GLCM_Entropy_log10	2.72	2.72	2.69	2.8	2.57	1.95	2.63	2.84	2.48	2.89	2.82
GLCM_Dissimilarity	7.57	8.09	2.58	9.89	11.6	25.7	3.53	3.87	4.88	5.88	5.09
GLRLM_SRE	0.964	0.975	0.905	0.975	0.983	0.984	0.954	0.942	0.954	0.959	0.959
GLRLM_LRE	1.15	1.12	1.57	1.13	1.1	1.09	1.23	1.27	1.21	1.18	1.18
GLRLM_LGRE	0.0116	0.00331	0.00186	0.00296	0.0121	0.0158	0.00288	0.00184	0.0151	0.00357	0.00396
GLRLM_HGRE	427	984	1100	993	1410	1300	804	1140	352	1050	660
GLRLM_SRLGE	0.0112	0.00326	0.00167	0.0029	0.0118	0.0155	0.00277	0.00176	0.0147	0.00341	0.00383
GLRLM_SRHGE	417	961	1010	965	1390	1280	771	1080	341	1010	632
GLRLM_LRLGE	0.013	0.00363	0.00295	0.00326	0.0144	0.0171	0.00343	0.00222	0.0169	0.00424	0.00456
GLRLM_LRHGE	470	1090	1640	1130	1540	1400	970	1440	398	1230	783
GLRLM_GLNU	31.9	27	437	64.2	16.6	5.67	75	356	21.5	54.4	100
GLRLM_RLNU	780	827	12400	2280	844	263	2290	10200	441	1730	2730
GLRLM_RP	0.953	0.965	0.87	0.966	0.976	0.977	0.939	0.923	0.938	0.945	0.946
NGLDM_Coarseness	0.00818	0.00813	0.00158	0.00475	0.0134	0.0371	0.00658	0.00102	0.019	0.00593	0.00347
NGLDM_Contrast	0.279	0.334	0.0763	0.241	0.642	0.694	0.107	0.128	0.208	0.2	0.151
NGLDM_Busyness	0.124	0.0702	0.353	0.127	0.041	0.0154	0.0967	0.444	0.0747	0.0928	0.195
GLZLM_SZE	0.699	0.745	0.53	0.739	0.814	0.817	0.67	0.665	0.69	0.697	0.682
GLZLM_LZE	6.78	4.39	656	3.99	2.78	2.58	11.4	66.5	11.7	10.6	12.7
GLZLM_LGZE	0.0135	0.00385	0.00192	0.0032	0.00851	0.0152	0.0033	0.00258	0.022	0.00356	0.00453
GLZLM_HGZE	523	1030	1320	951	1430	1350	882	1160	440	1100	651
GLZLM_SZLGE	0.0112	0.00332	0.000903	0.00251	0.00409	0.0106	0.0024	0.002	0.0197	0.00218	0.00334
GLZLM_SZHGE	410	783	712	691	1190	1140	619	774	324	779	444
GLZLM_LZLGE	0.0657	0.0118	1.29	0.00977	0.0447	0.0354	0.0267	0.0791	0.117	0.0285	0.032
GLZLM_LZHGE	1960	3960	411000	4480	3890	3180	7800	74600	2450	9180	9090
GLZLM_GLNU	15.5	15.8	80.6	42	12.6	4.01	33.2	113	8.63	27.9	48
GLZLM_ZLNU	219	292	894	797	400	127	527	1720	112	454	669
GLZLM_ZP	0.541	0.625	0.184	0.63	0.72	0.731	0.466	0.328	0.475	0.497	0.485

Patient	122	123	124	125	126
Type(GBM=2, AO=1)	1	1	1	1	1
minValue	543	1030	289	669	154
meanValue	814	2120	491	931	512
stdValue	136	356	89.5	130	95.2
maxValue	1490	3680	781	1360	735
HISTO_Skewness	1.04	0.135	0.433	0.833	-0.943
HISTO_Kurtosis	4.72	2.94	2.9	3.64	3.6
HISTO_Entropy_log10	1.52	1.54	1.65	1.65	1.59
HISTO_Energy	0.0362	0.0334	0.0258	0.027	0.0317
SHAPE_Volume (mL)	11.2	3.3	0.565	3.32	4.43
GLCM_Homogeneity	0.297	0.241	0.229	0.201	0.311
GLCM_Energy	0.00305	0.00325	0.00243	0.002	0.00223
GLCM_Contrast	89.9	116	82.5	145	37.7
GLCM_Correlation	0.454	0.118	0.727	0.539	0.829
GLCM_Entropy_log10	2.76	2.7	2.67	2.77	2.85
GLCM_Dissimilarity	6.42	8.09	6.99	9.2	4.51
GLRLM_SRE	0.946	0.958	0.977	0.981	0.948
GLRLM_LRE	1.4	1.71	1.1	1.09	1.24
GLRLM_LGRE	0.00777	0.00472	0.00527	0.00836	0.00144
GLRLM_HGRE	447	802	851	772	1690
GLRLM_SRLGE	0.00731	0.00458	0.00519	0.0082	0.00138
GLRLM_SRHGE	430	770	832	758	1590
GLRLM_LRLGE	0.0113	0.0101	0.00564	0.00989	0.00166
GLRLM_LRHGE	568	1300	932	834	2140
GLRLM_GLNU	161	115	14.9	29.9	133
GLRLM_RLNU	4040	3260	547	1060	3710
GLRLM_RP	0.924	0.943	0.969	0.974	0.931
NGLDM_Coarseness	0.00156	0.00176	0.0178	0.00736	0.00332
NGLDM_Contrast	0.177	0.242	0.307	0.388	0.131
NGLDM_Busyness	0.515	0.343	0.0423	0.104	0.0954
GLZLM_SZE	0.627	0.552	0.752	0.788	0.655
GLZLM_LZE	28.4	15.8	3.31	2.84	20.3
GLZLM_LGZE	0.00696	0.00322	0.00602	0.00647	0.00195
GLZLM_HGZE	598	869	877	792	1530
GLZLM_SZLGE	0.00385	0.00141	0.00506	0.00432	0.00151
GLZLM_SZHGE	428	517	682	641	969
GLZLM_LZLGE	0.276	0.094	0.0143	0.0327	0.0162
GLZLM_LZHGE	7270	11000	2710	2120	41500
GLZLM_GLNU	53.5	43.7	9.74	21.3	51.7
GLZLM_ZLNU	639	418	208	461	735
GLZLM_ZP	0.358	0.385	0.661	0.702	0.405
