# Supplementary material for: Radiomics-Based Machine Learning Technology Enables Better Differentiation Between Glioblastoma and Anaplastic Oligodendroglioma
Source: Front Oncol. 2019 Nov 5;9:1164. doi: 10.3389/fonc.2019.01164 (PMC6848260; doi:10.3389/fonc.2019.01164)
Supplement: Supplementary Material 2 — Results of Manny-Whitney U-test to examine the difference between two sets of data extraction. [file Data_Sheet_2.PDF]

Hypothesis Test Summary

	Null Hypothesis	Test	Sig.	Decision
1	The distribution of minValue is the same across categories of Type: GBM=2, AO=1.	Independent -Samples Mann-Whitney U Test	.778	Retain the null hypothesis.
2	The distribution of meanValue is the same across categories of Type: GBM=2, AO=1.	Independent -Samples Mann-Whitney U Test	.884	Retain the null hypothesis.
3	The distribution of stdValue is the same across categories of Type: GBM=2, AO=1.	Independent -Samples Mann-Whitney U Test	.372	Retain the null hypothesis.
4	The distribution of maxValue is the same across categories of Type: GBM=2, AO=1.	Independent -Samples Mann-Whitney U Test	.567	Retain the null hypothesis.
5	The distribution of HISTO_Skewness is the same across categories of Type: GBM=2, AO=1.	Independent -Samples Mann-Whitney U Test	.712	Retain the null hypothesis.
6	The distribution of HISTO_Kurtosis is the same across categories of Type: GBM=2, AO=1.	Independent -Samples Mann-Whitney U Test	.356	Retain the null hypothesis.
7	The distribution of HISTO_Energy is the same across categories of Type: GBM=2, AO=1.	Independent -Samples Mann-Whitney U Test	.634	Retain the null hypothesis.
8	The distribution of GLCM_Homogeneity is the same across categories of Type: GBM=2, AO=1.	Independent -Samples Mann-Whitney U Test	.600	Retain the null hypothesis.

Asymptotic significances are displayed. The significance level is .05.

Hypothesis Test Summary

	Null Hypothesis	Test	Sig.	Decision
**9**	The distribution of GLCM_Energy is the same across categories of Type: GBM=2, AO=1.	Independent-Samples Mann-Whitney U Test	.127	Retain the null hypothesis.
**10**	The distribution of GLCM_Contrast is the same across categories of Type: GBM=2, AO=1.	Independent-Samples Mann-Whitney U Test	.409	Retain the null hypothesis.
**11**	The distribution of GLCM_Correlation is the same across categories of Type: GBM=2, AO=1.	Independent-Samples Mann-Whitney U Test	.332	Retain the null hypothesis.
**12**	The distribution of GLCM_Dissimilarity is the same across categories of Type: GBM=2, AO=1.	Independent-Samples Mann-Whitney U Test	.449	Retain the null hypothesis.
**13**	The distribution of GLRLM_SRE is the same across categories of Type: GBM=2, AO=1.	Independent-Samples Mann-Whitney U Test	.741	Retain the null hypothesis.
**14**	The distribution of GLRLM_LRE is the same across categories of Type: GBM=2, AO=1.	Independent-Samples Mann-Whitney U Test	.892	Retain the null hypothesis.
**15**	The distribution of GLRLM_LGRE is the same across categories of Type: GBM=2, AO=1.	Independent-Samples Mann-Whitney U Test	.174	Retain the null hypothesis.

Asymptotic significances are displayed. The significance level is .05.

(continued)

Hypothesis Test Summary

	Null Hypothesis	Test	Sig.	Decision
**16**	The distribution of GLRLM_HGRE is the same across categories of Type: GBM=2, AO=1.	Independent-Samples Mann-Whitney U Test	.218	Retain the null hypothesis.
**17**	The distribution of GLRLM_SRLGE is the same across categories of Type: GBM=2, AO=1.	Independent-Samples Mann-Whitney U Test	.207	Retain the null hypothesis.
**18**	The distribution of GLRLM_SRHGE is the same across categories of Type: GBM=2, AO=1.	Independent-Samples Mann-Whitney U Test	.232	Retain the null hypothesis.
**19**	The distribution of GLRLM_LRLGE is the same across categories of Type: GBM=2, AO=1.	Independent-Samples Mann-Whitney U Test	.256	Retain the null hypothesis.
**20**	The distribution of GLRLM_LRHGE is the same across categories of Type: GBM=2, AO=1.	Independent-Samples Mann-Whitney U Test	.171	Retain the null hypothesis.
**21**	The distribution of GLRLM_GLNU is the same across categories of Type: GBM=2, AO=1.	Independent-Samples Mann-Whitney U Test	.808	Retain the null hypothesis.
**22**	The distribution of GLRLM_RLNU is the same across categories of Type: GBM=2, AO=1.	Independent-Samples Mann-Whitney U Test	.961	Retain the null hypothesis.

Asymptotic significances are displayed. The significance level is .05.

(continued)

Hypothesis Test Summary

	Null Hypothesis	Test	Sig.	Decision
**23**	The distribution of GLRLM_RP is the same across categories of Type: GBM=2, AO=1.	Independent-Samples Mann-Whitney U Test	.786	Retain the null hypothesis.
**24**	The distribution of NGLDM_Coarseness is the same across categories of Type: GBM=2, AO=1.	Independent-Samples Mann-Whitney U Test	.600	Retain the null hypothesis.
**25**	The distribution of NGLDM_Contrast is the same across categories of Type: GBM=2, AO=1.	Independent-Samples Mann-Whitney U Test	.497	Retain the null hypothesis.
**26**	The distribution of NGLDM_Busyness is the same across categories of Type: GBM=2, AO=1.	Independent-Samples Mann-Whitney U Test	.313	Retain the null hypothesis.
**27**	The distribution of GLZLM_SZE is the same across categories of Type: GBM=2, AO=1.	Independent-Samples Mann-Whitney U Test	.763	Retain the null hypothesis.
**28**	The distribution of GLZLM_LZE is the same across categories of Type: GBM=2, AO=1.	Independent-Samples Mann-Whitney U Test	.741	Retain the null hypothesis.
**29**	The distribution of GLZLM_LGZE is the same across categories of Type: GBM=2, AO=1.	Independent-Samples Mann-Whitney U Test	.361	Retain the null hypothesis.

Asymptotic significances are displayed. The significance level is .05.

(continued)

Hypothesis Test Summary

	Null Hypothesis	Test	Sig.	Decision
30	The distribution of GLZLM_HGZE is the same across categories of Type: GBM=2, AO=1.	Independent-Samples Mann-Whitney U Test	.361	Retain the null hypothesis.
31	The distribution of GLZLM_SZLGE is the same across categories of Type: GBM=2, AO=1.	Independent-Samples Mann-Whitney U Test	.946	Retain the null hypothesis.
32	The distribution of GLZLM_SZHGE is the same across categories of Type: GBM=2, AO=1.	Independent-Samples Mann-Whitney U Test	.567	Retain the null hypothesis.
33	The distribution of GLZLM_LZLGE is the same across categories of Type: GBM=2, AO=1.	Independent-Samples Mann-Whitney U Test	.741	Retain the null hypothesis.
34	The distribution of GLZLM_LZHGE is the same across categories of Type: GBM=2, AO=1.	Independent-Samples Mann-Whitney U Test	.303	Retain the null hypothesis.
35	The distribution of GLZLM_GLNU is the same across categories of Type: GBM=2, AO=1.	Independent-Samples Mann-Whitney U Test	.831	Retain the null hypothesis.

Asymptotic significances are displayed. The significance level is .05.

(continued)

Hypothesis Test Summary

	Null Hypothesis	Test	Sig.	Decision
**36**	The distribution of GLZLM_ZLNU is the same across categories of Type: GBM=2, AO=1.	Independent-Samples Mann-Whitney U Test	.985	Retain the null hypothesis.
**37**	The distribution of GLZLM_ZP is the same across categories of Type: GBM=2, AO=1.	Independent-Samples Mann-Whitney U Test	.854	Retain the null hypothesis.

Asymptotic significances are displayed. The significance level is .05.
