# Supplementary material for: Radiomics-Based Machine Learning Technology Enables Better Differentiation Between Glioblastoma and Anaplastic Oligodendroglioma
Source: Front Oncol. 2019 Nov 5;9:1164. doi: 10.3389/fonc.2019.01164 (PMC6848260; doi:10.3389/fonc.2019.01164)
Supplement: Supplementary Material 3 — Texture features selected with three different methods. Features selected by all three methods are labeled red. Features selected by two methods are labeled in yellow. LASSO, least absolute shrinkage and selection operator; GBDT, gradient boosting decision tree. [file Data_Sheet_3.PDF]

| Type of TA feature | TA feature name | Description |
|---|---|---|
| **Co-occurrence matrix (GLCM):** takes into account the arrangements of pairs of voxels to extract textural indices | Homogeneity | Homogeneity of grey-level voxel pairs |
| | Energy | Uniformity of grey-level voxel pairs. |
| | Correlation | Linear dependency of grey-levels in GLCM |
| | Contrast | Local variations in the GLCM |
| | Entropy | Randomness of grey-level voxel pairs |
| | Dissimilarity | Variation of grey-level voxel pairs |
| **Grey-Level Run Length Matrix (GLRLM)**: gives the size of homogenous grey-level runs for each | SRE (short-run emphasis) | Distribution of the short homogeneous runs in an image |
| | LRE (long-run emphasis) | Distribution of the long homogeneous runs in an image |
| | LGRE (low grey-level run emphasis) | Distribution of the low grey-level runs |
| | HGRE (high grey-level run emphasis) | Distribution of the high grey-level runs |
| | SRLGE (short-run low grey-level | Distribution of the short homogenous runs with low grey-levels |
| | SRHGE (short-run high grey-level | Distribution of the short homogenous runs with high grey-levels |
| | LRLGE (long-run low grey-level | Distribution of the long homogeneous runs with low grey-levels |
| | LRHGE (long-run high grey-level | Distribution of the long homogeneous runs with high grey-levels |
| | GLNUr (grey-level non-uniformity for | Non-uniformity of the grey-levels of the homogeneous runs |
| | RLNU (run length non-uniformity) | Length of the homogeneous runs |
| | RP (run percentage) | Homogeneity of the homogeneous runs |
| **Neighbourhood Grey-Level Different Matrix (NGLDM)**: corresponds to the difference of grey-level between one voxel and its 26 neighbourhoods in three dimensions | Coarseness | Level of spatial rate of change in intensity |
| | Contrast | Intensity difference between neighbouring regions |
| | Busyness | Spatial frequency of changes in intensity |
| **Grey-Level Zone Length Matrix (GLZLM)**: provides information on the size of homogenous zones for each grey-level in three dimensions | SZE (short-zone emphasis) | Distribution of the short homogeneous zones in an image |
| | LZE (long-zone emphasis) | Distribution of the long homogeneous zones in an image |
| | LGZE (low grey-level zone emphasis) | Distribution of the low grey-level zones |
| | HGZE (high grey-level zone emphasis) | Distribution of the high grey-level zones |
| | SZLGE (short-zone low grey-level emphas | Distribution of the short homogenous zones with low grey-levels |
| | SZHGE (short-zone high grey-level empha | Distribution of the short homogenous zones with high grey-levels |
| | LZLGE (long-zone low grey-level emphas | Distribution of the long homogenous zones with low grey-levels |
| | LZHGE (long-zone high grey-level empha | Distribution of the long homogenous zones with high grey-levels |
| | GLNUz (grey-level non-uniformity for zor | Non-uniformity of the grey-levels of the homogeneous zones |
| | RLNU (zone length non-uniformity) | Length of the homogeneous runs |
| | ZP (zone percentage) | Homogeneity of the homogeneous zones |
| | Sphericity | Measures how spherical a volume of interest is |
| | Compacity | Measures the degree to which the volume of interest is compact |
| Indices from Histogram: provides information derived from global histogram analysis | Skewness | Measures the asymmetry of the grey-level distribution in the histogram. |
| | Kurtosis | Measures whether the grey-level distribution is peaked or flat relative to a |
| | Entropy | Measures the randomness of the distribution |
| | Energy | Measures the uniformity of the distribution |
