# Supplementary figures and images for: Radiomics-Based Machine Learning Technology Enables Better Differentiation Between Glioblastoma and Anaplastic Oligodendroglioma
Source: Front Oncol. 2019 Nov 5;9:1164. doi: 10.3389/fonc.2019.01164 (PMC6848260; doi:10.3389/fonc.2019.01164)

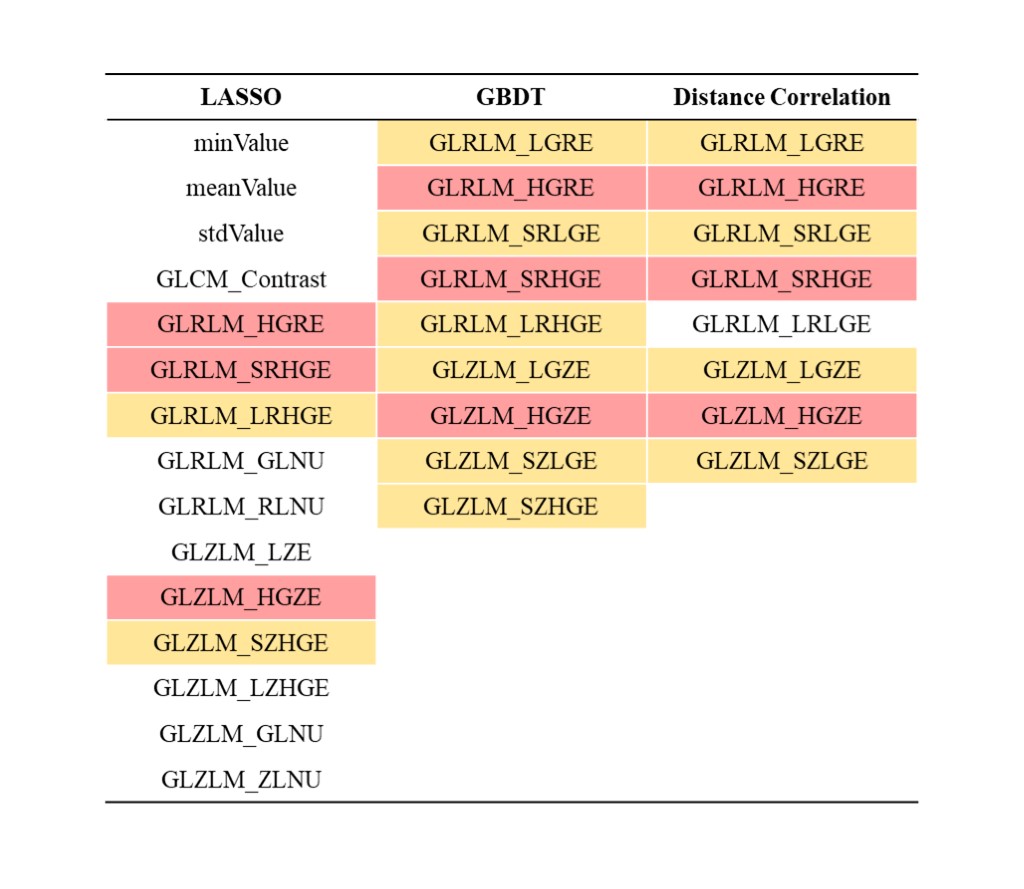

Supplement: Supplementary Material 4 — Explanation of each texture features. [file Image_1.JPEG]

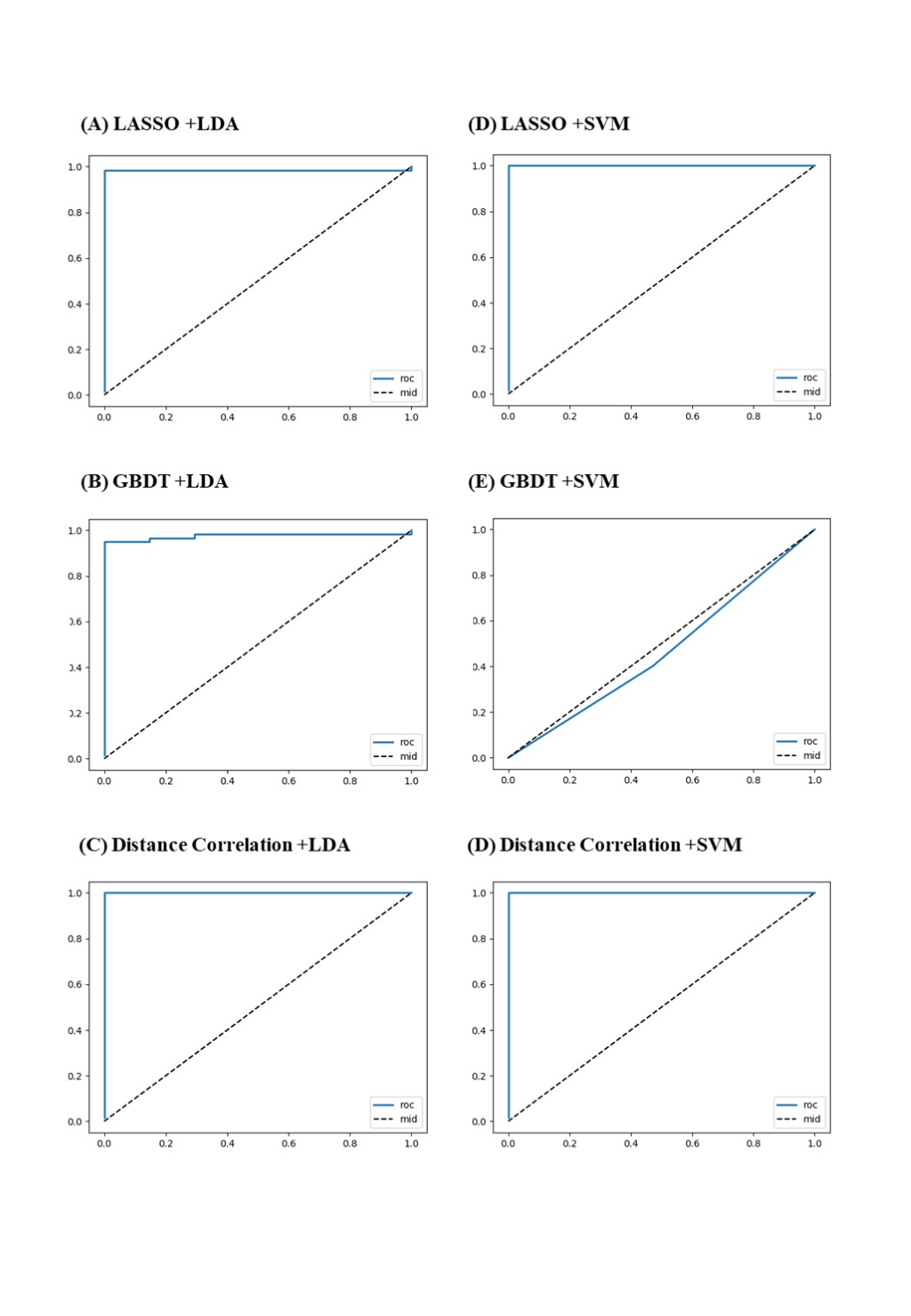

Supplement: Supplementary Material 5 — ROCs of six models to differentiate between GBM and AO. GBM, glioblastoma; AO, anaplastic oligodendroglioma; LASSO, least absolute shrinkage and selection operator; GBDT, gradient boosting decision tree; LDA, linear discriminant analysis; SVM, support vector machine. [file Image_2.JPEG]
